# Gene-Metabolite Networks of Volatile Metabolism in Airen and Tempranillo Grape Cultivars Revealed a Distinct Mechanism of Aroma Bouquet Production

**DOI:** 10.3389/fpls.2016.01619

**Published:** 2016-10-27

**Authors:** José L. Rambla, Almudena Trapero-Mozos, Gianfranco Diretto, Angela Rubio-Moraga, Antonio Granell, Lourdes Gómez-Gómez, Oussama Ahrazem

**Affiliations:** ^1^Facultad de Farmacia, Instituto Botánico, Universidad de Castilla-La ManchaAlbacete, Spain; ^2^Instituto de Biología Molecular y Celular de Plantas, CSIC-Universidad Politécnica de ValenciaValencia, Spain; ^3^Italian National Agency for New Technologies, Energy, and Sustainable Development, Casaccia Research CentreRome, Italy; ^4^Fundación Parque Científico y Tecnológico de Castilla-La ManchaAlbacete, Spain

**Keywords:** volatile organic compounds, precursors, aroma, expression analysis, *Vitis vinifera*

## Abstract

Volatile compounds are the major determinants of aroma and flavor in both grapes and wine. In this study, we investigated the emission of volatile and non-volatile compounds during berry maturation in two grape varieties (Airén and Tempranillo) throughout 2010 and 2011. HS-SPME coupled to gas chromatography and mass spectrometry was applied for the identification and relative quantitation of these compounds. Principal component analysis was performed to search for variability between the two cultivars and evolution during 10 developmental stages. Results showed that there are distinct differences in volatile compounds between cultivars throughout fruit development. Early stages were characterized in both cultivars by higher levels of some apocarotenoids such as β-cyclocitral or β-ionone, terpenoids (E)-linalool oxide and (Z)-linalool oxide and several furans, while the final stages were characterized by the highest amounts of ethanol, benzenoid phenylacetaldehyde and 2-phenylethanol, branched-amino acid-derived 3-methylbutanol and 2-methylbutanol, and a large number of lipid derivatives. Additionally, we measured the levels of the different classes of volatile precursors by using liquid chromatography coupled to high resolution mass spectrometry. In both varieties, higher levels of carotenoid compounds were detected in the earlier stages, zeaxanthin and α-carotene were only detected in Airén while neoxanthin was found only in Tempranillo; more variable trends were observed in the case of the other volatile precursors. Furthermore, we monitored the expression of homolog genes of a set of transcripts potentially involved in the biosynthesis of these metabolites, such as some glycosyl hydrolases family 1, lipoxygenases, alcohol dehydrogenases hydroperoxide lyases, O-methyltransferases and carotenoid cleavage dioxygenases during the defined developmental stages. Finally, based on Pearson correlation analyses, we explored the metabolite-metabolite fluctuations within VOCs/precursors during the berry development; as well as tentatively linking the formation of some metabolites detected to the expression of some of these genes. Our data showed that the two varieties displayed a very different pattern of relationships regarding the precursor/volatile metabolite-metabolite fluctuations, being the lipid and the carotenoid metabolism the most distinctive between the two varieties. Correlation analysis showed a higher degree of overall correlation in precursor/volatile metabolite-metabolite levels in Airén, confirming the enriched aroma bouquet characteristic of the white varieties.

## Introduction

Secondary metabolites of grapes (*Vitis vinifera L*.) play a key role in wine quality. The phenolic components of the skin and seeds are the main source of the color of wine and its structural properties (Ribereau-Gayon and Glories, 1986), while volatile organic compounds (VOCs) are the major determinants of aroma and flavor in wine (Zoecklein et al., 1998).

The final aroma of wine is determined by several hundreds of volatile compounds of varying chemical nature. Among these compounds, alcohols, esters, aldehydes, ketones, and hydrocarbons have been characterized, all at very low concentrations with a human threshold detection ranging between 10^−4^ and 10^−12^ g/L (Koundouras et al., 2009). The concentration of these compounds in the final product depends on factors associated with grape variety, cultivation (climate, irrigation, etc.) as well as the fermentation process (pH, temperature, nutrients and microflora) and posterior management involving factors such as filtration, clarification or aging. The characteristics and intensity of aroma may vary depending on the grape variety used, and also the geographical and climatic conditions where the grapes were grown. The volatile compounds that contribute to the aroma of the grape are mainly esters of acetic acid and modified monoterpenoids such as linalool, geraniol, nerol, citronellol, α-terpineol, and hotrienol (Rapp and Mandery, 1986). Other groups of volatile aromatic compounds playing an important role in aroma are aldehydes such as (E)-2-hexenal and hexanal, ketones, e.g., 2- and 3-alkanones; and alcohol compounds including n-alcohols from 4 to 11 carbon atoms, unsaturated alcohols and short-chain branched and aromatic alcohols such as benzyl alcohol. Furthermore, it should be noted that a large amount of compounds responsible for the aroma have been described in grape in glycosylated non-volatile form (Winterhalter and Skouroumounis, 1997). The most abundant included within this group are modified terpenes, particularly monoterpenes.

The existence of a non-volatile and odorless grape fraction that can be revealed by chemical or enzymatic pathways was first demonstrated by Cordonier and Bayonove (1974).

During the past two decades, a growing number of studies have shown that the glycosides represent a natural reservoir of volatile compounds in a high number of fresh or processed fruits (Buttery et al., 1990; Marlatt et al., 1992; Buttery, 1993; Krammer et al., 1994; Sakho et al., 1997; Boulanger and Crouzet, 2001; Aubert et al., 2003; Lalel et al., 2003; Osorio et al., 2003; Tikunov et al., 2010, 2013), in grapes and wine (Williams, 1993; Baek and Cadwallader, 1999; Genovés et al., 2003; Sarry and Günata, 2004), and also in flowers and plants and their derivatives (Loughrin et al., 1992; Straubinger et al., 1998; Wang et al., 2001; Watanabe et al., 2001; Nonier et al., 2005). The enzymatic and acid hydrolysis of glycosylated precursors release the volatile aglycones, thus changing the flavor (Williams, 1993). Enzymatic hydrolysis is catalyzed by glycosidases. This is a large group of biologically important enzymes, both biomedical and industrial, which are found in plants and microorganisms, mainly yeasts, and filamentous fungi (Pogorzelski and Wilkowska, 2007). Although, endogenous glycosidic activities increased in the fruit during the ripening process, no evidence of their relationship with the hydrolysis of glycosylated precursors of volatile compounds has been proved so far (Lecas et al., 1991; Kumar and Ramón, 1996; Manzanares et al., 2001; Mizutani et al., 2002; Sarry and Günata, 2004; Wei et al., 2004; Tsuruhami et al., 2006).

As stated before, in grape berries there are hundreds of compounds that potentially contribute to the aroma and flavor of wine. The nature of these metabolites shows a large chemical diversity and belongs to different metabolic pathways producing mainly fatty acids, amino acids, esters and terpenoid derivatives.

Volatiles derived from fatty acids are a class of compounds which includes one of the most important volatiles produced in many fruits. These compounds are classified as green leaf volatiles due to their characteristic “green” fresh aroma of cut grass, since high amounts of lipid-derived C_6_ aldehydes and alcohols are released from vegetative tissues when disrupted (Klee, 2010; Rambla et al., 2014). The initial step in the biosynthesis of these compounds is still not completely understood. Due to their toxicity free fatty acids are rapidly catabolized mainly by means of the lipoxygenase pathway which includes the sequential activity of lipoxygenase (LOX) and hydroperoxide lyase (HPL) enzymes (Tieman et al., 2012). The aldehydes produced from this LOX pathwaycan be reduced to alcohols by means of alcohol dehydrogenases (ADHs), enzymes catalyzing their reversible interconversion (Speirs et al., 1998a,b; Tesniere et al., 2006).

A variety of compounds are derived from the amino acid phenylalanine, such as 2-phenylethanol, phenylacetaldehyde and benzaldehyde, some of which provide a floral aroma (Baldwin et al., 2008; Tzin et al., 2013). The main biosynthesis of these compounds is started by means of a phenylalanine ammonia-lyase (PAL) producing (E)-cinnamic acid, while the last steps of the biosynthesis of some of these compounds are catalyzed by an O-methyltransferase (Mageroy et al., 2012).

Other important volatile compounds are terpenoids, which can be classified into two groups: monoterpenoids (C_10_) and sesquiterpenoids (C_15_). They are both synthesized from the five-carbon precursors isopentenyl diphosphate (IPP) and dimethylallyl diphosphate (DMAPP). The carotenoid derived volatiles, such as the C_13_ ketones β-ionone or β-damascenone, are synthesized by the oxidative cleavage of double bonds in carotenoids carried out by carotenoid cleavage dioxygenases (CCDs) (El Hadi et al., 2013; Granell and Rambla, 2013; Frusciante et al., 2014; Rubio-Moraga et al., 2014; Ahrazem et al., 2016).

Many of the volatiles are not preformed but produced by action of enzymes on precursors or conjugated substrates. To see to what extent the variability in volatile production from berries of two grape cultivars Airén (white) and Tempranillo (red) is in part due to differences in precursors levels or precursor availability to the volatile pathway, we study the profiles of volatiles and non-volatiles metabolites and investigated the correlations among the level of volatile compounds and their precursors and the expression of some genes potentially involved in their formation. A positive correlation between precursors and final volatile products will help us to exploit biotechnologically their potential to increase volatiles by selecting varieties with more precursors or conjugated forms of volatiles.

## Material and methods

### Plant material

Grapevine berries and leaves of healthy *Vitis vinifera* L. from Tempranillo and Airén varieties were sampled in Tarazona de la Mancha, Spain, during 2010 and 2011. The two genotypes are cultivated in neighboring vineyards thus they are under the same climatic, microclimatic and stress impacts. Vineyard management was carried out to provide optimum plant growth and yield including fertilization, plant protection treatment, irrigation and canopy management according to local viticulture standards.

For every 10 plants, three bunches of grapes were sampled over a 10-week period from the end of July to early October. A total of 10 samples corresponding to 10 different stages were inspected visually before sampling and only intact and healthy bunches were taken. The weekly samples corresponding to the phenology of the two cultivars is shown in Supplementary Figure 1. After collection, all samples were immediately frozen in liquid nitrogen and stored at −80°C until required.

### Volatile detection and quantification

For volatile analysis, three biological replicates were processed and analyzed independently for each developmental stage. Each biological replicate consisted in a pool of about 500 g of whole berries in the same developmental stage. Samples were cooled with liquid nitrogen, ground with mortar and pestle, and stored at −80°C until analysis. Prior to the analysis of volatile compounds, frozen fruit powder (1 g fresh weight) from each sample was weighed in a 7 mL vial, closed, and incubated at 30°C for 10 min. Then, 2.2 g of CaCl_2_.2H_2_O and 1 mL of EDTA 100 mM were added, shaken gently and sonicated for 5 min, and 1.5 mL of the homogenized mixture was transferred into a 10 ml screw cap headspace vial, where volatiles were collected from.

Volatile compounds were extracted by headspace solid-phase microextraction (HS-SPME) by means of a 65 μm PDMS/DVB fiber (Supelco). Initially, headspace vials were tempered at 50°C for 10 min. Then, the volatiles were extracted by exposing the fiber to the vial headspace for 30 min under continuous agitation and heating at 50°C. The extracted volatiles were desorbed in the GC injection port for 1 min at 250°C in splitless mode. Incubation of the vials, extraction and desorption were performed automatically by a CombiPAL autosampler (CTC Analytics). Chromatography was performed on a 6890N gas chromatograph (Agilent Technologies) with a DB-5ms (60 m × 0.25 mm × 1 μm) column (J&W Scientific) with Helium as carrier gas at a constant flow of 1.2 mL/min. Oven temperature conditions were: 40°C for 2 min, 5°C/min ramp until 250°C and then held at 250°C for 5 min. Mass spectra were recorded in scan mode in the 35–250 m/z range by a 5975B Mass Spectrometer (Agilent Technologies) at an ionization energy of 70 eV and a scanning speed of 6 scans/s. MS source temperature was 230°C. Chromatograms and spectra were recorded and processed using the Enhanced ChemStation software (Agilent Technologies).

For GC-MS, compounds were unequivocally identified by comparison of both mass spectrum and retention time to those of pure standards (SIGMA-Aldrich), except those labeled with an asterisk, which were tentatively identified by comparison of their mass spectra with those in the NIST05 library. For quantification, peak areas of selected specific ions were integrated for each compound and normalized by comparison with the peak area of the same compound in a reference sample injected regularly in order to correct for variations in detector sensitivity and fiber aging. The reference sample consisted in a homogeneous mixture of all the samples analyzed. Data for a particular sample were expressed as the relative content of each metabolite compared to those in the reference.

### Precursors detection and quantification by LC-MS

Carotenoids and chlorophylls have been analyzed and quantified by LC-DAD-APCI-HRMS as previously described (Liu et al., 2014) (Su et al., 2015) with slight modifications. Forty milligram of freeze-dried berry powder have been used for each extraction, and APCI-MS settings were as following: sheath and auxiliary gas, set to 30 and 12 units, respectively; the vaporizer temperature and the capillary temperature were set to 270 and 220°C, respectively, while the discharge current was set to 3.5 μA, and the capillary voltage and tube lens settings were 25 V and 80 V. Identification was performed using literature data (Mendes-Pinto et al., 2004; Crupi et al., 2010; Kamffer et al., 2010), and on the basis of the m/z accurate masses, as reported on Pubchem (http://pubchem.ncbi.nlm.nih.gov/) or Chemspider (http://www.chemspider.com). Linoleic and linolenic acids have been analyzed using the same experimental conditions, confirmed by using authentic standards, and have been relatively quantified as fold on the internal standard (α-tocopherol acetate) level. For each experimental point, at least 4 independent extractions have been used.

LC-ESI(+)-HRMS analysis of semi-polar precursors of volatiles (amino acids, phenylpropanoids, terpene glucosides) has been performed as previously described (De Vos et al., 2007; Iijima et al., 2008) with slight modifications. 20 mg of freeze-dried grape berry powder were extracted with 0.75 mL cold 75% (v/v) methanol, 0.1% (v/v) formic acid, spiked with 10 μg/ml formononetin. After shaking for 40′at 20 Hz using a Mixer Mill 300 (Qiagen), samples were centrifuged for 15 min at 20,000 g at 4°C. 0.6 mL of supernatant was removed and transfer to HPLC tubes. For each genotype/stage, at least five independent extractions have been carried out. LC-MS analyses were carried out using a LTQ-Orbitrap Discovery mass spectrometry system (Thermo Fisher Scientific) operating in positive electrospray ionization (ESI), coupled to an Accela U-HPLC system (Thermo Fisher Scientific, Waltham, MA). Liquid chromatography was carried out using a Phenomenex C18 Luna column (150 × 2.0 mm, 3 μm) and mobile phase was composed by water −0.1% Formic Acid (A) and acetonitrile −0.1% Formic Acid (B). The gradient was: 95%A:5%B (1 min), a linear gradient to 25%A:75%B over 40 min, 2 min isocratic, before going back to the initial LC conditions in 18 min. Ten microliter of each sample were injected and a flow of 0.2 mL was used during the whole LC runs. Detection was carried out continuously from 230 to 800 nm with an online Accela Surveyor photodiode array detector (PDA, Thermo Fischer Scientific, Waltham, MA). All solvents used were LC-MS grade quality (CHROMASOLV®from Sigma-Aldrich). Metabolites were quantified in a relative way by normalization on the internal standard amounts. ESI-MS ionization was performed using the following parameters: capillary voltage and temperature were set at 10V and 285°C; sheath and aux gas flow rate at, respectively, 40 and 10. Spray voltage was set to 6 kV and tube lens at 60 V. Metabolite identification was performed by through comparing chromatographic and spectral properties with authentic standards and reference spectra, literature data, and on the basis of the m/z accurate masses, as reported on Pubchem database (http://pubchem.ncbi.nlm.nih.gov/) for monoisotopic masses identification, or on Metabolomics Fiehn Lab Mass Spectrometry Adduct Calculator (http://fiehnlab.ucdavis.edu/staff/kind/Metabolomics/MS-Adduct-Calculator/) in case of adduct ion detection.

### RNA extraction and quantitative real-time PCR analysis

The same batch of material used for RNA extraction was used for volatiles analysis. Total RNA extractions were performed as reported (Gómez-Gómez et al., 2012). The quantitative RT-PCR was carried out on cDNA from three biological replicates; reactions were set up in GoTaq® qPCR Master Mix (Promega, Madison WI, USA) according to manufacturer's instructions, with gene-specific primers (0.125 μM) in a final volume of 25 μl. The grapevine Genoscope database was used to identify sequences related to GH, CCD, LOX, HPL, ADH, and OMT genes (http://www.genoscope.cns.fr/externe/GenomeBrowser/Vitis/). The Primer design was performed using Primer3 program (http://frodo.wi.mit.edu/) (Rozen and Skaletsky, 2000). Primer sequences are listed in Supplementary Table 1. Transcripts were normalized to a reference number derived from transcript levels of the constitutively expressed 18rRNA. The cycling parameters of qPCR consisted of an initial denaturation at 94°C for 5 min; 40 subsequent cycles of denaturation at 94°C for 20 s, annealing at 58°C for 20 s and extension at 72°C for 20 s; and final extension at 72°C for 5 min. Assays were conducted with a StepOne™ Thermal Cycler (Applied Biosystems, California, USA) and analyzed using StepOne software v2.0 (Applied Biosystems, California, USA). Analyses of qRT-PCR data used the classic (1 + E)^−ΔΔCT^ method (C_T_ is the threshold cycles of one gene, E is the amplification efficiency). ΔC_T_ is equal to the difference in threshold cycles for target (X) and reference (R) (C_T, X_-C_T, R_), while the ΔΔC_T_ is equal to the difference of ΔC_T_ for stage 1 (C) and the other stages (T) (ΔC_T, T_-ΔC_T, C_) for each variety. The amplification system (e.g., primer and template concentrations) was properly optimized, and the efficiency was close to 1. So the amount of target, normalized to an endogenous reference and relative to a calibrator, is given by: Amount of target = 2^−ΔΔCT^. The qPCR products were separated on a 1.0% agarose gel and, then, were sequenced to confirm their identity using an automated DNA sequencer (ABI PRISM 3730xl, Perkin Elmer) from Macrogen Inc. (Seoul, Korea). Additionally, subsequent reactions for DNA melt curves were created for each primer combination to confirm the presence of a single product.

### Statistical and bioinformatics analysis

For Principal Component Analysis (PCA), the complete dataset including all replicates was considered. The ratio of the signal relative to a reference consisting in a homogeneous mixture of all the samples was used, after log2 transformation. PCA was performed by means of the program SIMCA-P version 11 (Umetrics, Umea, Sweden) with Unit Variance normalization.

Pearson correlation coefficients were calculated with SPSS version 15.0 software (SPSS Inc., Chicago, USA) with the relative target quantity in samples based on the comparative C_T_ (ΔΔC_T_) method of each gene and the log 2 transformed levels of the average ratio of each volatile/precursor metabolite for each variety and developmental stage. A Hierarchical Cluster Analysis (HCA) was performed with the resulting correlation values using the Acuity 4.0 program (Axon Instruments), with the distance measures based on Pearson correlation. Data from the correlation matrix were represented as a heatmap or correlation network by means of the Acuity 4.0 program.

Gene and metabolite data were transformed in linear fold change and Pearson correlation coefficients (|ρ|) were calculated using the PAST software (http://folk.uio.no/ohammer/past/). Subsequently, gene-metabolite correlation heat maps and matrices were built and colored using the GENE-E software (http://www.broadinstitute.org/cancer/software/GENE-E/). Finally, correlation networks were performed as previously described (Diretto et al., 2010).

## Results and discussion

### Volatile profiling

Airén and Tempranillo, sourced from Castilla-La Mancha (Spain), represent important commercial varieties in this region. A study was carried out over a 2-year period to determine the evolution of the volatile fractions and also some of their precursors during grape development. In addition, we have tentatively associated some of the genes potentially involved in their formation expressions and the metabolites found in different stages of development in both varieties.

Many studies have sought to analyse and characterize wine VOCs in Tempranillo (Rosillo et al., 1999; Hermosín Gutiérrez, 2003; González et al., 2007; Izquierdo Cañas et al., 2008; López et al., 2008; Cynkar et al., 2010) and Airén (Gonzalez-Viñas et al., 1996; Pérez-Coello et al., 1999; Rosillo et al., 1999; Pérez-Coello et al., 2000; Castro Vázquez et al., 2002; Hernández-Orte et al., 2005). However, few studies have been dedicated to the aroma of grape juices in Tempranillo (González-Mas et al., 2009) or Airén (García et al., 2003).

There are clear and distinct aroma differences between grape cultivars, which are due to differences in their profile of volatile compounds. These variations are mostly attributed to differences in the levels of the substances that constitute the aroma of grape rather than to qualitative differences in the volatile compounds produced. Headspace solid phase microextraction (HS-SPME) technique was chosen for the acquisition of volatiles due to its high sensitivity and low manipulation required.

A total of 55 compounds were identified in the volatile fraction of both Airén and Tempranillo. Forty-eight of these metabolites were unequivocally identified by both mass spectra and retention index with those of authentic standards. For the other 7 compounds, a tentative identification based on their mass spectra similarity was proposed.

The volatiles identified are detailed in Table 1, which also shows the nature of the compounds detected in the 10 developmental stages analyzed for the two cultivars (Airén and Tempranillo) and their classification into different metabolic pathways: phenylpropanoids, terpenoids, lipid derivatives, branched-chain amino acid and other amino acid derivatives and norisoprenoids. Most of the VOCs shown in Table 1 had been previously detected in these varieties (González-Mas et al., 2009; Cejudo-Bastante et al., 2011). The complete results obtained for the analysis of volatiles of all the samples are shown in Supplementary Table 2.

**Table 1:** **Volatile compounds detected in Tempranillo and Airén fruit juice**.

**ID**	**Compound**	**Identification**	**Compound nature**	**Metabolic pathway**
1	Acetaldehyde	Unequivocal	Aldehyde	
2	Ethanol	Unequivocal	Alcohol	
3	Acetone	Unequivocal	Ketone	
4	2-propanol	Unequivocal	Alcohol	
5	Ethyl ether	Unequivocal	Ether	
6	2-methyl-2-propanol	Unequivocal	Alcohol	BAA?
7	Ethyl acetate	Unequivocal	Ester	
8	2-methyl-2-butanol	Unequivocal	Alcohol	BAA?
9	1-butanol	Unequivocal	Alcohol	
10	1-penten-3-ol	Unequivocal	Alcohol	L
11	Pentanal	Unequivocal	Aldehyde	L
12	2-ethylfuran	Unequivocal	Furan	L?
13	3-methylbutanol	Unequivocal	Alcohol	BAA
14	2-methylbutanol	Unequivocal	Alcohol	BAA
15	(E)-2-pentenal	Unequivocal	Aldehyde	L
16	1-pentanol	Unequivocal	Alcohol	L
17	(Z)-3-hexenal	Unequivocal	Aldehyde	L
18	Hexanal	Unequivocal	Aldehyde	L
19	(E)-2-hexenal	Unequivocal	Aldehyde	L
20	(E)-2-hexen-1-ol	Unequivocal	Alcohol	L
21	1-hexanol	Unequivocal	Alcohol	L
22	Heptanal	Unequivocal	Aldehyde	L
23	(E,E)-2,4-hexadienal	Unequivocal	Aldehyde	L
24	(E)-2-heptenal	Unequivocal	Aldehyde	L
25	1-octen-3-ol	Unequivocal	Alcohol	L
26	2-pentylfuran	Unequivocal	Furan	L?
27	2-octanol	Unequivocal	Alcohol	L
28	(E,E)-2,4-heptadienal	Unequivocal	Aldehyde	L
29	Limonene	Unequivocal	Monoterpene	T
30	Benzylalcohol	unequivocal	Alcohol/phenolic	Ph
31	Unknown 25.22 (furan)	Tentative	Furan	
32	Phenylacetaldehyde	Unequivocal	Aldehyde/phenolic	AA
33	(E)-2-octenal	Unequivocal	Aldehyde	L
34	Salicylaldehyde	Unequivocal	Aldehyde/phenolic	Ph
35	1-octanol	Unequivocal	Alcohol	L
36	(Z)-linalool oxide	Unequivocal	Monoterpenoid	T
37	(E)-linalool oxide	Unequivocal	Monoterpenoid	T
38	2-ethylhexanoic acid	Unequivocal	Organic acid	
39	Linalool	Unequivocal	Monoterpenoid	T
40	2-phenylethanol	Unequivocal	Alcohol/phenolic	AA
41	(E)-2-nonenal	Unequivocal	Aldehyde	L
42	3-ethylbenzaldehyde	Unequivocal	Aldehyde/phenolic	Ph?
43	2-ethylbenzaldehyde	Unequivocal	Aldehyde/phenolic	Ph?
44	Terpineol	Unequivocal	Monoterpenoid	T
45	Methyl salicylate	Unequivocal	Ester/phenolic	Ph
46	β-cyclocitral	Unequivocal	Norisoprenoid	C
47	Nonanoic acid	Unequivocal	Organic acid	L
48	Unkown 33.56 (apocarotenoid)	Tentative	Norisprenoid	C
49	2, 6-diisocyanatotoluene	Tentative	Phenolic	
50	beta-damascenone	Unequivocal	Norisprenoid	C
51	Ylangene	Tentative	Sesquiterpene	T
52	β-ionone	Unequivocal	Norisprenoid	C
53	Unknown 39.38 (sesquiterpene)	Tentative	Sesquiterpene	T
54	1-(2,3,6-trimethylphenyl)-3-buten-2-one	Tentative	Ketone/phenolic	
55	Unknown 39.96 (sesquiterpene)	Tentative	Sesquiterpene	T

*BAA, branched-amino acid derivative; L, lipid derivative; Ph, phenylpropanoid; AA, other amino acid derivatives; T, terpenoid; C, apocarotenoid. A question mark indicates some degree of uncertainty about the metabolic pathway*.

**Figure 1:**
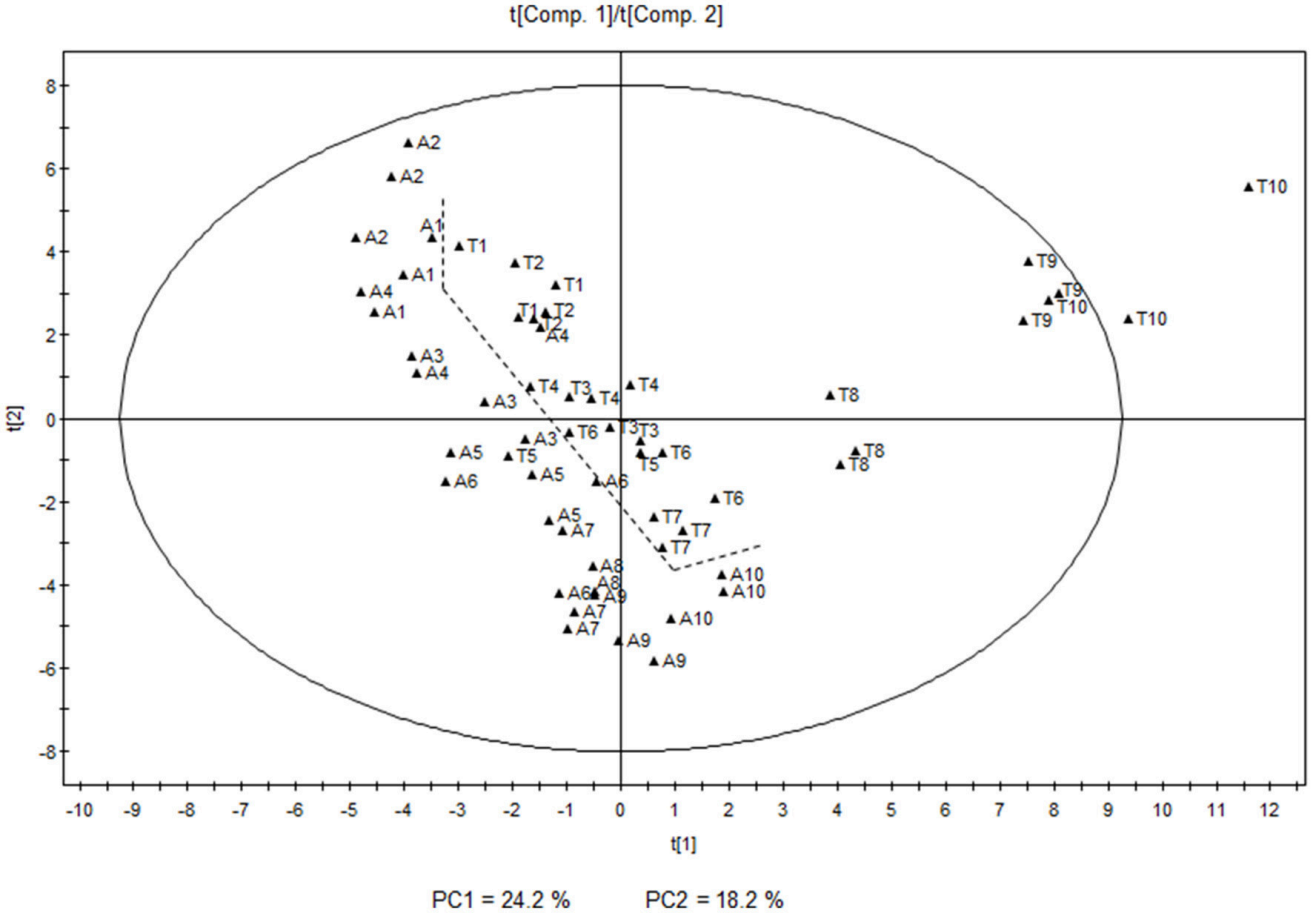
**Principal Component Analysis score plot showing the individual samples of the 10 developmental stages analyzed in both Airén (A) and Tempranillo (T) varieties (1, earliest; 10, latest) for season 2011**. Principal components 1 and 2 have been represented. The dashed line shows the separation between the two varieties.

Principal Component Analysis (PCA) was performed in order to unravel the main features of the differences in volatile compounds among the samples. The score plot of the first two principal components, explaining 24.2 and 18.2% of the total variability respectively, separates the two varieties throughout the different stages based on their distinct volatile profiles. It can also be appreciated that there is a parallel evolution of the volatile levels during most of the developmental stages in both varieties, with the exception of the latest stages in Tempranillo, when a dramatic change is developed and a characteristic profile appears (Figure 1).

The volatile profile of Tempranillo was characterized by higher levels of branched-chain amino acid-derived compounds such as 3-methylbutanol, 2-methylbutanol, 2-methyl-2-propanol and 2-methyl-2-butanol, C_5_ lipid derivatives pentanal, 1-pentanol, 1-penten-3-ol and (E)-2-pentenal, other lipid derivatives such as 1-hexanol, heptanal (only detected in this variety), (E)-2-heptenal, (E,E)-2,4-heptadienal, 1-octen-3-ol, (E)-2-octenal and (E)-2-nonenal, phenylpropanoids methyl salicylate, salicylaldehyde, benzylalcohol, phenylacetaldehyde, and 2-phenylethanol, which has been reported to be important in the flavor of Noble muscadine wine, and also other compounds such as 2-pentylfuran, acetaldehyde, ethyl ether, and ethyl acetate.

**Figure 2:**
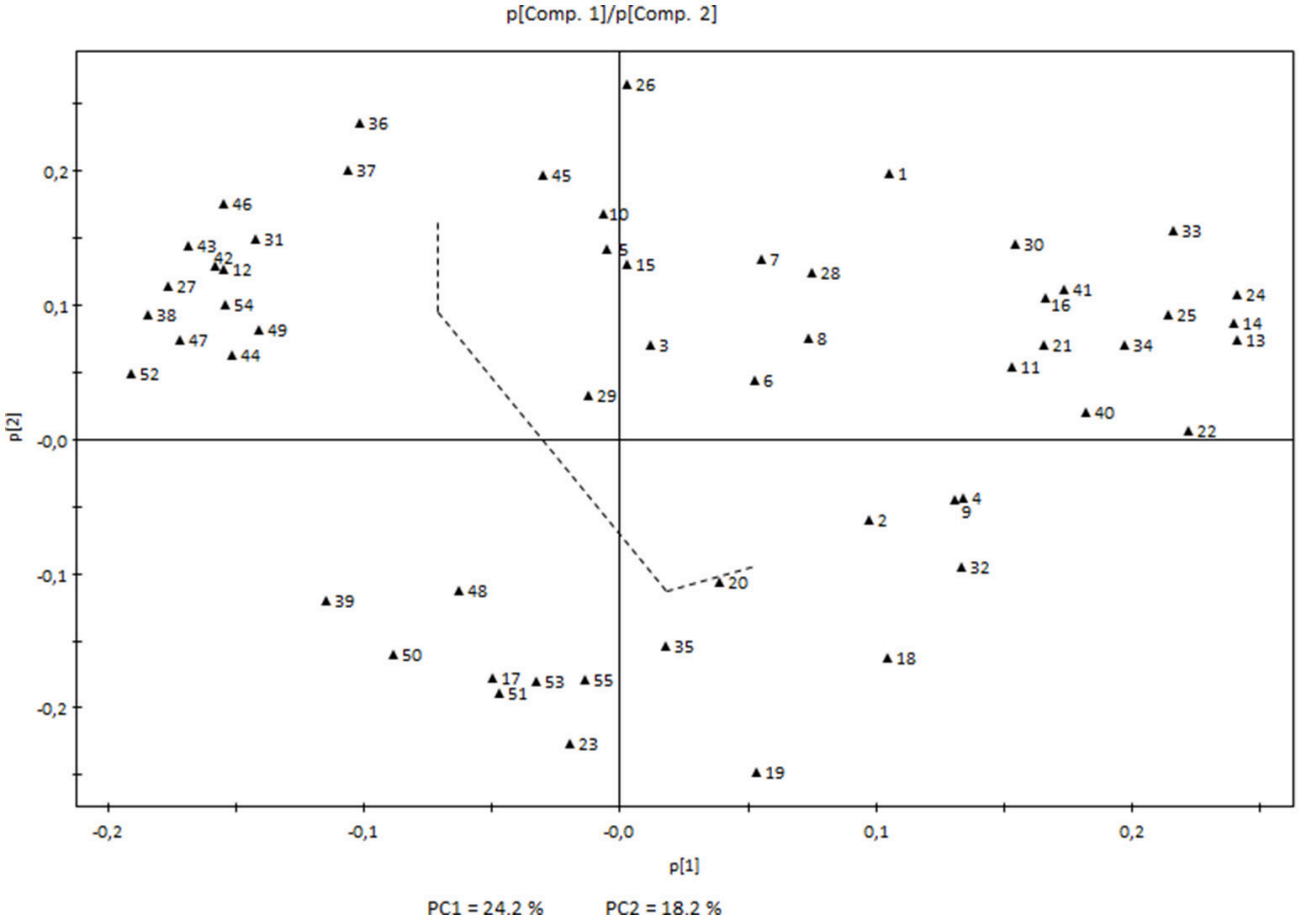
**Principal Component Analysis loading plot showing the volatile compounds determining the first two principal components**. The two components represented and the dashed line correspond to those in the score plot (Figure 1). Each number corresponds to a volatile compound as indicated in Table 1 and Supplementary Table 2.

On the other hand, white variety Airén showed higher levels of norisoprenoids (β-damascenone, β-ionone, β-cyclocitral, and an unknown compound at 33.56 min with a norisoprenoid structure), monoterpenoids (linalool, terpineol and (Z)- and (E)-linalool oxide isomers), sesquiterpenes (ylangene and two unidentified compounds at 39.38 and 39.96 min only detected in this variety), C_6_ lipid-derived aldehydes ((Z)-3-hexenal, (E)-2-hexenal and (E,E)-2,4-hexadienal) and also a few other compounds characteristic of early developmental stages (Figure 2).

Regarding the contribution to aroma of apocarotenoids, we know from the sensory work done that β-damascenone and β-ionone have a very low perception threshold. Thus, β-ionone confers intense fruity and floral aromas with violet notes (Silva Ferreira and Guedes De Pinho, 2004). The main characteristics of these volatile profiles, with minor variations, were consistent in the 2 years of sampling.

Lipid derivatives are generated by the enzymatic degradation of lipids when cellular tissue breakdown occurs (Baldwin et al., 1998), while their level in the intact fruit is minimal (Riley and Thompson, 1998). This applies, for example, to C_6_ aldehydes (hexanal and (Z)-3-hexenal) responsible for the “green” flavor note, cut grass aroma and fruity notes of grapes (Vilanova et al., 2010). Hexanal and (Z)-3-hexenal are formed, respectively, from linoleic and linolenic acids, after the action of a lipoxygenase (LOX) followed by a hydroperoxide lyase (HPL) (Granell and Rambla, 2013). Hexanal and (Z)-3-hexenal can then be reduced to their corresponding alcohols, 1-hexanol and (Z)-3-hexenol, respectively, by the action of an alcohol dehydrogenase (ADH) (Galliard et al., 1977). The instability of (Z)-3-hexenal has been described in many papers (Buttery et al., 1987, 1988), and especially its broad isomerization to (E)-2-hexenal (Galliard et al., 1977; Buttery, 1993; Riley and Thompson, 1998; Gray et al., 1999).

Norisoprenoids are obtained from the degradation of carotenoids such as β-carotene or lycopene (Stevens, 1970; Simkin et al., 2004; Lewinsohn et al., 2005b). These reactions take place only in the fruit, and in some cases occur after cellular tissue breakdown. However, the biochemical nature of these degradative oxidation mechanisms, and the enzymes and genes involved should be reviewed in each case (Lewinsohn et al., 2005a).

Compounds which originate from amino acids can be subsequently converted into aldehydes, alcohols and esters, via various steps including deamination, decarboxylation, reduction and esterification (Crouzett, 1992). Phenylpropanoid volatile compounds such as 2-phenylethanol, methyl salicylate, or benzaldehyde are primarily derived from phenylalanine. The synthesis of some of these compounds requires the shortening of the carbon skeleton side chain by a C_2_ unit, which can potentially occur via either the β-oxidative pathway or non-oxidatively (Dudareva et al., 2004; Orlova et al., 2006; Pichersky et al., 2006).

C_6_ aldehydes are partially responsible for the green, herbaceous and sometimes bitter aromas of wine. Volatile phenolic compounds such as phenylacetaldehyde figure as one of the substances responsible for the hyacinth and rose-like odor described in the French-Romanian Admira grape variety (Wang and Kays, 2000), while terpenoid and norisoprenoid compounds are responsible for the floral and fruity aroma of wine made from these varieties.

As previously mentioned, a clear evolution in the volatile profile throughout grape development was also observed. This was markedly parallel in both varieties, except for the last ripening stages in Tempranillo, where a dramatic change was detected between stages 7 and 8–10. The score plot shows the existence of four different stages for both cultivars corresponding to immature, unripe, ripe and over-ripe. Higher levels of carotenoid derivatives were characteristic of the earlier stages of development in both varieties with higher levels of some apocarotenoids such as β-cyclocitral or β-ionone, and monoterpenoids (E)-linalool oxide, (Z)-linalool oxide, and terpineol. Higher levels of other compounds such as 2-ethylbenzaldehyde, 3-ethylbenzaldehyde, 1-(2,3,6-trimethylphenyl)-3-buten-2-one, 2-octanol, and 2-ethylhexanoic acid were also characteristic in these stages. Carotenoids may undergo breakdown reactions that generate C_13_ norisoprenoid compounds involved in the typical aromas of some grapevine cultivars (Baumes et al., 2002). Apocarotenoids are mostly generated by the cleavage of a carotenoid molecule by enzymes of the CCD family.

In the last stages of development in Tempranillo (T8-T10), a dramatic change in the volatile profile was observed, characterized by a significant increase in the levels of branched-chain amino acid-derived alcohols 3-methylbutanol and 2-methylbutanol, a set of lipid-derived aldehydes ((E)-2-heptenal, (E)-2-octenal, (E)-2-nonenal, heptanal, and pentanal) and alcohols (1-octen-3-ol, 1-hexanol, and 1-pentanol), and some phenylpropanoid compounds (salicylaldehyde, benzyl alcohol, and 2-phenylethanol).

The last stages of development were not characterized by such a dramatic change in Airén volatiles; although a significant increase in lipid-derived compounds such as hexanal, (E)-2-hexenal, (E)-2-hexen-1-ol, and 1-octanol was observed (Figure 2).

### Volatile precursors profiling

In order to search for possible volatile precursors, we carried out LC-HRMS analyses of the non-volatile fractions (both polar and non-polar by, respectively, ESI- and APCI-MS) in both varieties. A total of 56 compounds were identified in the Airén and Tempranillo samples, belonging to different metabolic pathways. Forty-eight of these metabolites were unequivocally identified by retention index and maximal absorption wavelength with those of authentic standards. Eight compounds were annotated as unknown from which 6 were carotenoids and 2 were chlorophylls (Tables 2A,B).

**Table 2A:** **Volatile precursors detected in Airén throughout maturation stages**.

**Metabolites**	**λmax**	**RT**	**A1**	**A2**	**A3**	**A4**	**A5**	**A6**	**A7**	**A8**	**A9**	**A10**
**AMINO ACIDS**												
Isoleucine		1.29	0.0171	0.0166	0.0036	0.0096	0.0094	0.0065	0.0116	0.0036	0.0061	0.0103
Leucine		1.82	0.0327	0.0378	0.0140	0.0313	0.0369	0.0296	0.0404	0.0175	0.0300	0.0453
Phenylalanine^*^		2.77	0.0624	0.1002	0.0688	0.2004	0.1131	0.0722	0.1144	0.0628	0.0753	0.1733
Valine		1.25	0.0138	0.0134	0.0034	0.0089	0.0090	0.0092	0.0056	0.0054	0.0091	0.0132
**LIPIDS**												
Linoleic Acid		0.90	0.0064	0.0037	0.0059	0.0051	0.0072	0.0044	0.0060	0.0053	0.0032	0.0041
Linolenic Acid		0.70	0.0452	0.0464	0.0559	0.0426	0.0284	0.0313	0.0235	0.0215	0.0159	0.0258
**CAROTENOIDS**												
Unknown carotenoid (1)	(375) 400, 421, 441	1.15	0.0604	0.0131	0.0006	0.0022	NF	NF	NF	NF	NF	NF
Unknown carotenoid (2)	(375) 400, 421, 441	1.34	0.0944	0.0877	0.0001	0.0017	NF	NF	NF	NF	NF	NF
Unknown carotenoid (3)	398, 422, 444	1.52	0.0213	0.0095	0.0016	NF	0.0005	NF	NF	0.0004	NF	NF
Unknown carotenoid (4)	421, 444, 470	1.70	0.1252	0.0093	NF	0.0023	NF	NF	NF	NF	NF	NF
Unknown carotenoid (5)	(375) 400, 421, 441	1.83	0.0843	0.0335	0.0000	0.0008	0.0023	NF	NF	NF	NF	NF
Neochrome-like structure	400, 421, 445	2.00	0.0364	0.0100	0.0015	NF	NF	0.0005	0.0091	0.0008	NF	0.0001
Neochrome a	401, 419, 446	2.27	0.0334	0.0144	0.0044	0.0113	0.0009	0.0016	NF	0.0021	0.0010	0.0016
Neoxanthin	420, 440, 466	2.40	NF	NF	NF	NF	NF	NF	NF	NF	NF	NF
Neochrome b	402, 421, 447	2.63	0.0062	0.0071	0.0054	0.0177	NF	NF	0.0055	0.0041	0.0016	0.0035
Luteoxanthin isomer	402, 421, 447	2.84	0.0115	0.0269	0.0106	0.0201	0.0044	0.0046	0.0209	0.0087	0.0053	NF
All-trans-violaxanthin	416, 442, 463	2.87	NF	NF	NF	NF	NF	NF	NF	NF	NF	NF
9-cis-violaxanthin	438, 462	2.91	NF	NF	NF	NF	0.0148	0.0431	0.0050	0.0010	0.0096	0.0166
Luteoxanthin	421, 444, 465	3.05	NF	NF	NF	NF	NF	NF	NF	NF	NF	NF
Auroxanthin	403, 425, 444	3.13	0.0033	0.0054	0.0023	0.0047	0.0020	0.0315	0.0072	0.0003	0.0018	0.0051
Flavoxanthin	(375), 402, 423, 446	3.47	0.0115	0.0068	0.0028	0.0510	NF	0.0132	0.0200	0.0048	0.0006	0.0015
All-trans-lutein	445, 472	4.09	0.3771	0.9528	1.1306	0.9413	0.1485	0.5769	0.6059	0.1842	0.2535	0.1136
Zeaxanthin	450, 476	4.77	0.4398	0.2700	0.2641	0.0000	0.0786	0.0656	0.1452	NF	0.1565	0.0739
13Z or 13Z′ lutein	442, 468	4.87	NF	0.0128	0.0091	0.0601	0.0046	0.0060	0.0022	0.0775	0.0072	0.0066
9Z or 9Z′ lutein	438, 467	4.98	NF	NF	NF	0.0022	NF	NF	0.0020	0.0026	0.0007	0.0013
Lutein-like structure	424, 446, 473	5.14	0.0605	0.0695	NF	NF	NF	NF	NF	NF	NF	NF
9Z or 9Z′ lutein-like structure	440, 468	5.45	NF	NF	0.0120	0.0821	0.0107	0.0143	0.0241	0.0276	0.0209	0.0053
Lutein-like structure	424, 446, 473	5.50	0.2215	0.2220	0.0288	0.0249	0.0238	NF	0.0472	NF	0.0154	0.0059
5, 8-epoxy-β-carotene	406, 426, 450	6.29	0.0639	0.0879	0.0225	0.0762	0.0179	0.0180	0.0115	NF	NF	0.0119
All-trans-α-carotene	444, 470	6.42	0.0349	0.0495	0.0117	0.0373	0.0224	0.0142	0.0038	0.0153	0.0123	0.0167
All-trans-β-carotene	452, 477	7.01	0.1263	0.3116	0.3068	0.2428	0.0698	0.1008	0.2784	0.0908	0.1184	0.0616
9-cis-β-carotene	423, 447, 473	7.13	0.0655	0.0991	0.0762	0.0660	0.0250	0.0230	0.0478	0.0208	0.0365	0.0275
Unknown carotenoid (6)	422, 446	7.75	0.0056	0.0100	0.0101	NF	NF	NF	0.0091	NF	NF	NF
All-trans-δ-carotene	453	8.02	0.0572	0.0295	0.0046	NF	NF	NF	0.0110	NF	NF	NF
TOTAL			1.9402	2.3385	1.9058	1.6446	0.4263	0.9134	1.2561	0.4412	0.6413	0.3528
**CHLOROPHYLLS**												
Pyropheophorbide b	436, 655	2.08	NF	NF	NF	NF	NF	NF	NF	NF	NF	NF
Pyropheophorbide a	410, 665	2.22	0.0010	NF	NF	NF	NF	NF	NF	NF	NF	0.0003
Chlorophyll b	469, 651	3.73	NF	NF	NF	NF	0.1866	0.0530	0.1466	NF	0.1687	0.2337
Chlorophyll a	432, 665	5.05	NF	NF	NF	NF	NF	NF	NF	NF	NF	NF
Unknown chlorophyll derivative (1)	401	5.64	0.3015	0.4567	0.0850	0.0805	0.0445	NF	0.0940	NF	0.0024	NF
Unknown chlorophyll derivative (2)	402	6.04	0.0222	0.0080	0.0050	0.0048	0.0031	0.0055	0.0117	0.0065	0.0038	NF
Pheophytin a-like (1)	410	6.12	0.1240	0.0854	0.0589	0.0047	NF	NF	NF	NF	NF	NF
Pheophytin a-like (2)	415, 445, 665	6.39	0.0168	0.0135	0.0141	0.0066	NF	0.0015	0.0045	0.0011	NF	0.0007
Pheophytin b-like	419, 436, 656	6.52	0.0286	0.0107	0.0048	0.0059	NF	NF	0.0071	0.0139	NF	NF
Pheophytin b	432, 654	6.89	0.5318	0.8759	0.4672	0.7853	0.0533	0.1730	0.2664	0.3316	0.1672	0.1702
Pheophytin a	408, 666	6.91	0.6346	0.9565	0.1426	0.1744	0.1006	0.1075	0.2378	0.3304	0.0841	0.0347
TOTAL			1.6604	2.4068	0.7776	1.0622	0.3881	0.3406	0.7681	0.6835	0.4262	0.4396
**OTHERS**												
α-tocopherol	290	2.74	1.0555	4.4248	3.1581	6.5971	2.0164	0.9774	3.6324	1.0689	1.9921	0.7909
Ubiquinone	296	5.89	9.3522	7.8888	4.0349	4.9211	2.6044	1.6224	6.2746	3.6986	5.0433	2.7090
Unknown	255	6.28	5.0114	12.8318	1.0207	10.8884	27.9940	5.7073	5.4368	4.9682	22.1602	4.9485
TOTAL			15.4192	25.1455	8.2137	22.4066	32.6148	8.3071	15.3438	9.7357	29.1956	8.4484
**PHENYLPROPANOIDES**												
Benzoic acid		10.84	0.0033	0.0054	0.0005	0.0005	0.0007	0.0005	0.0005	0.0001	0.0003	0.0002
Caffeic acid		7.36	0.0001	0.0001	NF	NF	NF	NF	NF	NF	NF	NF
Cinnamic acid		13.45	0.0001	0.0002	0.0002	0.0005	0.0003	0.0002	0.0003	0.0002	0.0002	0.0005
Coniferyl acetate		18.20	0.0005	0.0005	0.0005	0.0006	0.0005	0.0007	0.0264	0.0016	0.0008	0.0010
Coniferyl alcohol		0.72	0.0009	0.0008	0.0004	0.0004	0.0004	0.0004	0.0005	0.0004	0.0003	0.0004
Coniferyl aldehyde		10.56	0.0005	0.0005	NF	NF	NF	NF	NF	NF	NF	NF
Coumaric acid		8.86	0.0040	0.0040	0.0012	0.0011	0.0017	0.0018	0.0022	0.0015	0.0017	0.0020
Ferulic acid		10.59	0.0002	0.0003	NF	NF	NF	NF	NF	NF	NF	NF
Hydroxyconiferyl alcohol		6.56	0.0002	0.0002	NF	NF	NF	NF	0.0002	NF	NF	NF
Sinapyl alcohol		0.80	NF	NF	NF	NF	NF	NF	NF	NF	NF	NF
**TERPENES**												
α-terpinol-[xylosyl-(1->6)-glucoside]		7.25	0.0005	0.0002	0.0001	0.0001	0.0001	0.0001	0.0003	0.0001	0.0001	0.0002
α-terpinol-beta-d-glucoside		9.00	0.0002	0.0001	NF	NF	NF	NF	NF	NF	NF	NF
L-Linalool 3-[xylosyl-(1->6)-glucoside]		9.76	0.0012	0.0010	0.0003	0.0002	0.0002	0.0002	0.0005	0.0002	0.0001	0.0003
Limonene-arabinofuranose		11.83	0.0001	0.0002	0.0001	0.0002	0.0001	0.0001	0.0002	0.0002	0.0001	0.0002
Limonene-arabinofuranose-glucoside		15.62	0.0000	0.0000	0.0000	0.0001	NF	0.0000	0.0001	0.0001	0.0000	0.0001
Linalool-arabinofuranose		10.09	0.0001	0.0001	NF	NF	NF	NF	NF	NF	NF	NF
Linalyl-beta-d-glucoside		9.20	0.0004	0.0003	NF	NF	NF	NF	NF	NF	NF	NF
E-/Z-linalool oxide-arabinofuranose1^**^		9.00	0.0010	0.0006	NF	NF	NF	NF	NF	NF	NF	NF
E-/Z-linalool oxide-arabinofuranose2^**^		11.78	0.0001	0.0001	NF	NF	NF	NF	NF	NF	NF	NF
E-/Z-linalool oxide-arabinofuranose-glucoside1^**^		9.71	0.0013	0.0010	0.0003	0.0002	0.0002	0.0002	0.0004	0.0001	0.0001	0.0003
E-/Z-linalool oxide-arabinofuranose-glucoside2^**^		10.09	0.0007	0.0006	0.0001	0.0001	0.0001	0.0001	0.0003	0.0001	0.0000	0.0001
E-/Z-linalool oxide-glucoside1^**^		7.80	0.0002	0.0002	NF		NF	NF	0.0001	NF	NF	0.0001
E-/Z-linalool oxide-glucoside2^**^		9.00	0.0002	0.0001	NF	NF	NF	NF	NF	NF	NF	NF
E-/Z-linalool oxide-rhamnopyranose1^**^		8.10	0.0004	0.0003	NF	0.0000	NF	NF	NF	NF	NF	NF
E-/Z-linalool oxide-rhamnopyranose2^**^		9.50	0.0002	0.0001	NF	NF	NF	NF	NF	NF	NF	NF
E-/Z-linalool oxide-Rhamnopyranoside-glucoside^**^		12.08	0.0001	0.0002	0.0001	NF	0.0001	0.0000	0.0001	NF	NF	NF
E-/Z-linalool oxide-Rhamnopyranoside-glucoside2^**^		12.65	0.0000	0.0000	0.0000	NF	0.0000	0.0000	0.0000	NF	NF	0.0000

*Isoprenoids (carotenoids, chlorophylls, ubiquinone, and a-tocopherol) were identified and quantified by LC-DAD-APCI-MS and data are expressed as μg/mg DW.Other metabolite precursors were detected and relatively quantified by LC-APCI-MS (lipids) and LC-ESI-MS (amino acids, phenylpropanoids, terpene glucosides) and data are expressed as fold on the internal standard level (APCI, a-tocopherol acetate; ESI, formononetin). For more details, see materials and methods*.

* *Also involved in phenylpropanoid-derived volatiles*.

** *Not possible discriminating the E- and Z- isomers*.

**Table 2B:** **Volatile precursors detected in Tempranillo throughout maturation stages**.

**Metabolites**	**λmax**	**RT**	**T1**	**T2**	**T3**	**T4**	**T5**	**T6**	**T7**	**T8**	**T9**	**T10**
**AMINO ACIDS**												
Isoleucine			0.0054	0.0045	0.0128	0.0016	0.0083	0.0033	0.0020	0.0228	0.0104	0.0032
Leucine			0.0167	0.0173	0.0519	0.0072	0.0443	0.0175	0.0102	0.1005	0.0596	0.0153
Phenylalanine^*^			0.0478	0.0578	0.1588	0.0255	0.1013	0.0457	0.0404	0.1215	0.1178	0.0368
Valine			0.0128	0.0097	0.0230	0.0039	0.0138	0.0066	0.0039	0.0364	0.0220	0.0059
**LIPIDS**												
Linoleic Acid			0.0098	0.0089	0.0086	0.0057	0.0056	0.0076	0.0076	0.0112	0.0064	0.0056
Linolenic Acid			0.0444	0.0850	0.0767	0.0286	0.0822	0.0232	0.0344	0.4349	0.0582	0.0372
**CAROTENOIDS**												
Unknown carotenoid (1)	(375) 400, 421, 441	1.15	0.0786	0.0457	0.1030	NF	NF	NF	NF	NF	NF	NF
Unknown carotenoid (2)	(375) 400, 421, 441	1.34	NF	NF	NF	NF	NF	NF	NF	NF	NF	NF
Unknown carotenoid (3)	398, 422, 444	1.52	0.0561	0.0595	0.0569	0.0244	0.0188	0.0131	0.0064	NF	NF	NF
Unknown carotenoid (4)	421, 444, 470	1.70	0.0175	NF	NF	NF	NF	NF	NF	NF	NF	NF
Unknown carotenoid (5)	(375) 400, 421, 441	1.83	NF	NF	0.0300	0.0284	0.0182	0.0165	0.0049	NF	0.1232	NF
Neochrome-like structure	400, 421, 445	2.00	0.1990	NF	NF	NF	NF	NF	NF	0.0189	0.1600	NF
Neochrome a	401, 419, 446	2.27	NF	NF	NF	NF	NF	NF	NF	NF	NF	NF
Neoxanthin	420, 440, 466	2.40	NF	NF	NF	NF	NF	NF	0.1006	NF	0.1230	NF
Neochrome b	402, 421, 447	2.63	0.0867	0.0686	0.0503	NF	NF	NF	NF	NF	0.0115	0.0709
Luteoxanthin isomer	402, 421, 447	2.84	0.3063	0.2396	0.0970	0.3509	0.0790	NF	NF	NF	0.0689	NF
All-trans-violaxanthin	416, 442, 463	2.87	NF	NF	NF	NF	NF	0.2267	0.2435	NF	NF	NF
9-cis-violaxanthin	438, 462	2.91	NF	0.1184	NF	NF	NF	0.1511	NF	NF	NF	NF
Luteoxanthin	421, 444, 465	3.05	NF	NF	0.0299	0.0247	0.0137	0.0554	0.1522	NF	0.3048	1.2015
Auroxanthin	403, 425, 444	3.13	0.3277	0.1742	0.0646	NF	NF	NF	NF	0.0619	0.1520	NF
Flavoxanthin	(375), 402, 423, 446	3.47	0.7499	0.2313	0.1284	NF	NF	NF	NF	0.1691	0.1482	NF
All-trans-lutein	445, 472	4.09	9.9916	7.6905	4.9760	4.4097	2.2627	2.9679	2.8582	1.9456	2.6001	2.7767
Zeaxanthin	450, 476	4.77	NF	NF	NF	NF	NF	NF	NF	NF	NF	NF
13Z or 13Z′ lutein	442, 468	4.87	0.9707	0.2728	0.1881	0.4108	0.0538	0.2203	NF	0.1092	0.1751	1.0202
9Z or 9Z′ lutein	438, 467	4.98	0.0866	0.1354	0.2069	0.0162	NF	0.1210	NF	NF	NF	NF
Lutein-like structure	424, 446, 473	5.14	0.0352	NF	NF	NF	NF	NF	NF	NF	NF	NF
9Z or 9Z′ lutein-like structure	440, 468	5.45	0.5244	0.6207	0.7687	0.6037	0.2918	0.1278	0.1456	0.2171	0.3323	0.5097
Lutein-like structure	424, 446, 473	5.50	0.3054	NF	NF	NF	NF	NF	NF	NF	NF	NF
5,8-epoxy-β-carotene	406, 426, 450	6.29	0.9041	0.5826	0.5256	0.3810	0.0945	0.2507	0.1520	0.1829	0.1075	NF
All-trans-α-carotene	444, 470	6.42	NF	NF	NF	NF	NF	NF	NF	NF	NF	NF
All-trans-β-carotene	452, 477	7.01	4.9373	3.8749	0.6841	1.4960	0.8937	1.3463	1.5620	0.9064	0.9788	0.2520
9-cis-β-carotene	423, 447, 473	7.13	1.7399	1.1741	0.6765	0.5374	0.2493	0.3821	0.3580	0.2368	0.3233	NF
Unknown carotenoid (6)	422, 446	7.75	0.0771	0.0364	0.0296	NF	NF	NF	NF	NF	NF	NF
All-trans-δ-carotene	453	8.02	0.0279	0.0426	0.0164	NF	NF	NF	NF	NF	NF	NF
TOTAL			21.4219	15.3672	8.6320	8.2832	3.9756	5.8788	5.5834	3.8481	5.6087	5.8310
**CHLOROPHYLLS**												
Pyropheophorbide b	436, 655	2.08	NF	NF	NF	NF	NF	NF	NF	0.0192	NF	NF
Pyropheophorbide a	410, 665	2.22	NF	NF	NF	NF	NF	NF	NF	0.0276	0.0912	NF
Chlorophyll b	469, 651	3.73	NF	2.6581	NF	2.7103	0.9423	3.1888	3.6151	NF	1.0158	2.2874
Chlorophyll a	432, 665	5.05	NF	NF	NF	NF	NF	0.0474	0.4265	NF	NF	NF
Unknown chlorophyll derivative (1)	401	5.64	1.0691	0.2496	0.3093	NF	NF	NF	0.0205	NF	NF	NF
Unknown chlorophyll derivative (2)	402	6.04	0.1963	0.0974	0.1222	NF	NF	NF	0.0186	0.0705	0.0444	NF
Pheophytin a-like (1)	410	6.12	0.1168	0.0516	0.1256	NF	0.0577	NF	NF	0.0256	0.0258	0.0198
Pheophytin a-like (2)	415, 445, 665	6.39	0.0698	NF	0.0585	NF	0.0390	NF	NF	NF	NF	NF
Pheophytin b-like	419, 436, 656	6.52	0.8094	0.4350	0.8440	0.2006	0.1131	NF	NF	0.2263	0.1325	NF
Pheophytin b	432, 654	6.89	14.1734	7.8599	11.3427	3.8593	3.0228	0.4781	0.4265	2.3711	3.3644	1.4385
Pheophytin a	408, 666	6.91	5.3704	3.2120	3.4230	2.0915	1.0261	1.0585	0.7032	0.6567	1.1197	0.5704
TOTAL			21.8052	14.5636	16.2253	8.8616	5.2010	4.7727	5.2103	3.3968	5.7939	4.3161
**OTHERS**												
α-tocopherol	290	2.74	45.1689	28.6563	12.3871	22.0931	13.4224	21.0568	19.4971	19.3449	25.3264	11.9777
Ubiquinone	296	5.89	27.9983	25.4948	18.1442	8.6920	NF	21.2234	13.5892	6.9020	19.9027	NF
Unknown	255	6.28	119.4096	NF	93.9746	NF	NF	NF	NF	54.0751	NF	NF
TOTAL			192.5768	54.1511	124.5060	30.7851	13.4224	42.2802	33.0863	80.3219	45.2291	11.9777
**PHENYLPROPANOIDES**												
Benzoic acid			0.0095	0.0053	0.0034	0.0006	0.0002	0.0002	0.0002	0.0001	0.0001	0.0001
Caffeic acid			NF	NF	NF	NF	NF	NF	NF	NF	NF	NF
Cinnamic acid			0.0001	0.0001	0.0005	0.0001	0.0003	0.0001	0.0001	0.0004	0.0004	0.0001
Coniferyl acetate			0.0008	0.0006	0.0006	0.0006	0.0006	0.0007	0.0007	0.0006	0.0006	0.0005
Coniferyl alcohol			0.0006	0.0003	0.0003	0.0001	NF	0.0004	NF	NF	NF	NF
Coniferyl aldehyde			0.0016	0.0007	0.0002	0.0003	0.0001	0.0004	0.0003	0.0001	NF	NF
Coumaric acid			0.0040	0.0030	0.0021	0.0017	0.0012	0.0013	0.0009	0.0014	0.0012	0.0010
Ferulic acid			0.0018	0.0009	NF	0.0003	NF	NF	NF	NF	NF	NF
Hydroxyconiferyl alcohol			0.0003	0.0002	0.0001	0.0001	0.0001	0.0001	0.0001	0.0001	NF	NF
Sinapyl alcohol			0.0007	0.0003	NF	0.0001	NF	NF	0.0002	NF	NF	NF
**TERPENES**												
α-terpinol-[xylosyl-(1->6)-glucoside]			0.0003	0.0002	0.0001	0.0002	0.0001	0.0002	0.0002	0.0001	0.0002	0.0002
α-terpinol-beta-d-glucoside			NF	NF	NF	NF	NF	NF	NF	NF	NF	NF
L-Linalool 3-[xylosyl-(1->6)-glucoside]			0.0003	0.0002	0.0001	0.0000	0.0000	0.0002	0.0000	0.0000	0.0000	0.0000
Limonene-arabinofuranose			0.0000	0.0002	0.0001	0.0001	0.0001	0.0001	0.0003	0.0002	0.0001	0.0002
Limonene-arabinofuranose-glucoside			NF	NF	NF	NF	NF	NF	NF	NF	NF	NF
Linalool-arabinofuranose			NF	NF	NF	NF	NF	NF	NF	NF	NF	NF
Linalyl-beta-d-glucoside			NF	NF	NF	NF	NF	NF	NF	NF	NF	NF
E-/Z-linalool oxide-arabinofuranose1^**^			0.0007	0.0004	NF	NF	NF	NF	NF	NF	NF	NF
E-/Z-linalool oxide-arabinofuranose2^**^			0.0002	0.0002	NF	NF	NF	NF	NF	NF	NF	NF
E-/Z-linalool oxide-arabinofuranose-glucoside1^**^			0.0004	0.0002	0.0001	0.0001	0.0000	0.0002	0.0000	NF	NF	NF
E-/Z-linalool oxide-arabinofuranose-glucoside2^**^			0.0002	0.0001	0.0001	NF	NF	NF	NF	NF	NF	NF
E-/Z-linalool oxide-glucoside1^**^			NF	NF	NF	NF	NF	NF	NF	NF	NF	NF
E-/Z-linalool oxide-glucoside2^**^			NF	NF	NF	NF	NF	NF	NF	NF	NF	NF
E-/Z-linalool oxide-rhamnopyranose1^**^			NF	NF	NF	NF	NF	NF	NF	NF	NF	NF
E-/Z-linalool oxide-rhamnopyranose2^**^			NF	NF	NF	NF	NF	NF	NF	NF	NF	NF
E-/Z-linalool oxide-Rhamnopyranoside-glucoside^**^			0.0001	0.0000	0.0001	0.0000	NF	0.0000	0.0000	0.0000	0.0000	0.0000
E-/Z-linalool oxide-Rhamnopyranoside-glucoside2^**^			0.0000	NF	0.0000	0.0000	NF	0.0000	NF	NF	0.0000	0.0000

*Isoprenoids (carotenoids, chlorophylls, ubiquinone, and a-tocopherol) were identified and quantified by LC-DAD-APCI-MS and data are expressed as μg/mg DW; other metabolite precursors were detected and relatively quantified by LC-APCI-MS (lipids) and LC-ESI-MS (amino acids, phenylpropanoids, terpene glucosides) and data are expressed as fold on the internal standard level (APCI, a-tocopherol acetate; ESI, formononetin). For more details, see materials and methods*.

* *Also involved in phenylpropanoid-derived volatiles*.

** *Not possible discriminating the E- and Z- isomers*.

A strong relationship has been reported between the amino acid profile of grape varieties and the relative levels of the higher alcohols in wine and therefore the final aroma in wine (Hernández-Orte et al., 2002). The four amino acids detected in Airén and Tempranillo varieties were isoleucine, leucine, valine, and phenylalanine. Phenylalanine was the most abundant amino acid in grapes from both cultivars. This aromatic amino acid has been reported to produce aromatic alcohols such as 2-phenylethanol (Rossouw et al., 2009), which has a characteristic honey/spice/rose/lilac aroma (Francis and Newton, 2005) and is considered to play an important role in white wine aroma (López et al., 2003). Different accumulation patterns were observed in red and white varieties. In Tempranillo, higher levels were detected at T8 stage for isoleucine, leucine and valine, while phenylalanine had its maximum accumulation at T3 stage, whereas in Airén, isoleucine and phenylalanine were higher at the earlier stages of maturation decreasing later on, and leucine and valine were abundant in both earlier and latest stages. These patterns were in agreement with those obtained by (Garde-CerdáN et al., 2009) where higher accumulation at the end of ripening of amino acids were found in red varieties (Monastrell organic, Syrah, and Merlot grapes), whereas in the white grape Petit Verdot it diminished at the same stage.

The C_6_ aldehydes and alcohols derived from fatty acids constitute the major aroma derivatives responsible for the “green” aroma and are generally formed by the action of lipoxygenase (LOX), hydroperoxide lyase (HPL), (3Z)-(2E) enal isomerase, and alcohol dehydrogenase (ADH) enzymes when the grape is crushed (Baldwin, 2002; Schwab et al., 2008). Lipidic precursors linoleic and linolenic acids were detected in all the samples. Linoleic acid was detected at very low levels in Airén, while its levels in Tempranillo were about 25-fold higher. Linolenic acid was more abundant than linoleic acid in both varieties, and its levels were slightly higher in Tempranillo. In Airén, both fatty acids showed their higher levels in earlier stages, decreasing thereafter. A different pattern was observed in Tempranillo, with a sharp increase at stage T8, particularly dramatic in the case of linolenic acid. It is known that the phenolic content of grape is dependent on grape variety and maturity but is also influenced by variations in water and nutrient availability, light and temperature environment, and changes in predation and disease stresses (Downey et al., 2006; Cohen and Kennedy, 2010; Robinson et al., 2014). Phenylpropanoid precursors levels were found to be higher in Tempranillo than in Airén. In both varieties, benzoic acid and coumaric acid were found to be the predominant phenolic acids and were detected at the earlier stages. Benzoic acid is the precursor of several common hydroxybenzoic acids usually found in wine, such as gallic acid, gentisic acid, p-hydroxybenzoic acid, protocatechuic acid (3,4-dihydroxybenzoic acid), syringic acid, salicylic acid and vanillic acid (Peña et al., 2000; Pozo-Bayón et al., 2003; Monagas et al., 2005), whereas coumaric acid is a polyphenol precursor, especially for flavonoids, flavones and flavonols (Hrazdina et al., 1984). The latter acid is equally a crucial substrate for enzymes to create resveratrol (Goldberg et al., 1998). Sinapyl alcohol, one of the substrates necessary for the polymerization reactions that produce lignin, was only detected in Tempranillo, while caffeic acid was only found in Airén. The majority of phenylpropanoid precursors have a double sigmoid curve accumulation pattern with higher contents at the earlier and latest stages, a patterns which had been found for a variety of Semillon as well as for the flesh of Muscat Gordo Blanco berries (Francis et al., 1992).

Despite the diverse range of structures that have been isolated from natural sources, few carotenoids have been detected in grapes, 85% of the total carotenoids are β-carotene and lutein, with neochrome, neoxanthin, violaxanthin, luteoxanthin, flavoxanthin, lutein-5,6-epoxide and zeaxanthin, cis isomers of lutein and α-carotene the next most abundant (Mendes-Pinto, 2009). In both varieties, the carotenoids precursors were detected mainly at the earlier stages, with some exception where the metabolites were detected in all stages as all-trans lutein. Some of these metabolites were variety specific as the unknown carotenoid (2), all-tran-α-carotene, zeaxanthin and neochrome which were detected only in Airén, whereas neoxanthin, all-trans-violaxanthin and luteoxanthin were identified in Tempranillo. The concentration of some of these metabolites was higher in one of the varieties than the other; this is the case of the unknown carotenoid (4), which appears to be 4-fold higher in the red cultivar. Our data are in concordance with numerous studies on the evolution of carotenoids during grape development that pointed out that levels of β-carotene, lutein, flavoxanthin, and neoxanthin decrease drastically after veraison until maturation (Razungles et al., 1996; Bureau et al., 1998; Yuan and Qian, 2016). Processes of bioconversion of these compounds into others have been reported as, for example, the formation of violaxanthin from β-carotene as a consequence of the activation of the xanthophylls cycle at the end of maturation (Düring, 1999). Violaxanthin, lutein 5,6-epoxide and luteoxanthin only appear when higher concentration of sugar is reached, while neochrome is characteristic of green grapes (Guedes De Pinho et al., 2001; Grimplet et al., 2007; Deluc et al., 2009).

Regarding chlorophyll metabolites, chlorophyll a was absent under our analysis conditions, which could be due to the coefficient response of chlorophyll a, which is 4 times lower than the coefficient response of chlorophyll b, whereas pyropheohorbide b was only detected in Tempranillo. Higher concentrations of chlorophyll metabolites were detected in Tempranillo than in Airén. Chlorophyll-derived compounds are degraded more quickly than lutein or α-carotene. No chlorophyll-derived compounds are present in wines; grapes with a high content of these compounds are transformed into wines with a higher aromatic complexity (Winterhalter and Rouseff, 2002; Mendes-Pinto et al., 2005).

Other metabolites such as α-tocopherol, ubiquinone and an unknown metabolite with a maximum absorption at 290, 296, and 255, respectively, have been detected in both varieties and were present during all maturation stages. α-tocopherol is the main tocopherol detected in grape berries compared to γ and δ-tocopherols, while β-tocopherol was not found in the berries. Among the tocopherols present in foods, the α-homolog shows the highest vitamin E activity, thus making it the most important for human health and biological activity (Baydar, 2006; El Gengaihi et al., 2013).

Monoterpenes and sesquiterpenes play important roles in a number of different grape varieties as contributors to the overall aroma. Red grapes are not characterized by high levels of terpenes; however some terpenes are usually present at low levels (Canuti et al., 2009).

A total number of 17 glycosylated terpenes were detected in the samples analyzed. The total amounts of glycosylated terpenes were higher in Airén than in Tempranillo and 8 of these precursors were detected only in the white variety. In general, precursors with a disaccharide remained mainly constant throughout the maturation stages in both varieties while the precursors with one linked sugar were only found in stages 1 and 2, with the exception of limonene-arabinofuranose which was detected throughout all the stages in Airén.

The data obtained for glycosylated terpenes and carotenoids are in accordance with the levels detected for related volatile compounds, showing the highest levels of terpenoid and apocarotenoid volatiles in early stages and in the white variety Airén. On the contrary, the abundance of branched-chain amino acids and fatty acids do not seem to be in accordance to the levels observed for their related volatile compounds, likely due to the cell demand for keeping high contents in primary metabolites involved in a broad range of reactions and metabolic pathways.

### Metabolite-metabolite and metabolite-gene correlation analyses

In order to explore precursor/volatile metabolite-metabolite fluctuations during the ripening of the Airén and Tempranillo berries, we generated two correlation matrices (Supplementary Figure 2) by calculating the Pearson correlation coefficients for each data pair. Overall, the two varieties displayed a very different extent of relationships, stronger in Airén compared to Tempranillo. However, for both varieties we could identify a common “positive correlation core,” represented by the precursor/volatile metabolites involved in secondary pathways: phenylpropanoids, carotenoids, terpenes, and chlorophyll. This finding suggests the existence of a metabolic co-regulation during berry ripening, implying a general decrease in compounds which are exploited as volatile precursors, or are associated to early developmental stages. Oppositely, primary metabolism, particularly in lipids, exhibited a very distinct attitude between the two varieties: Airén berries showed a general negative correlation between lipids against the secondary metabolites, which was not observed in the Tempranillo matrix, indicating a different contribution of the lipid metabolism in the generation of the berry aroma bouquet.

**Figure 3:**
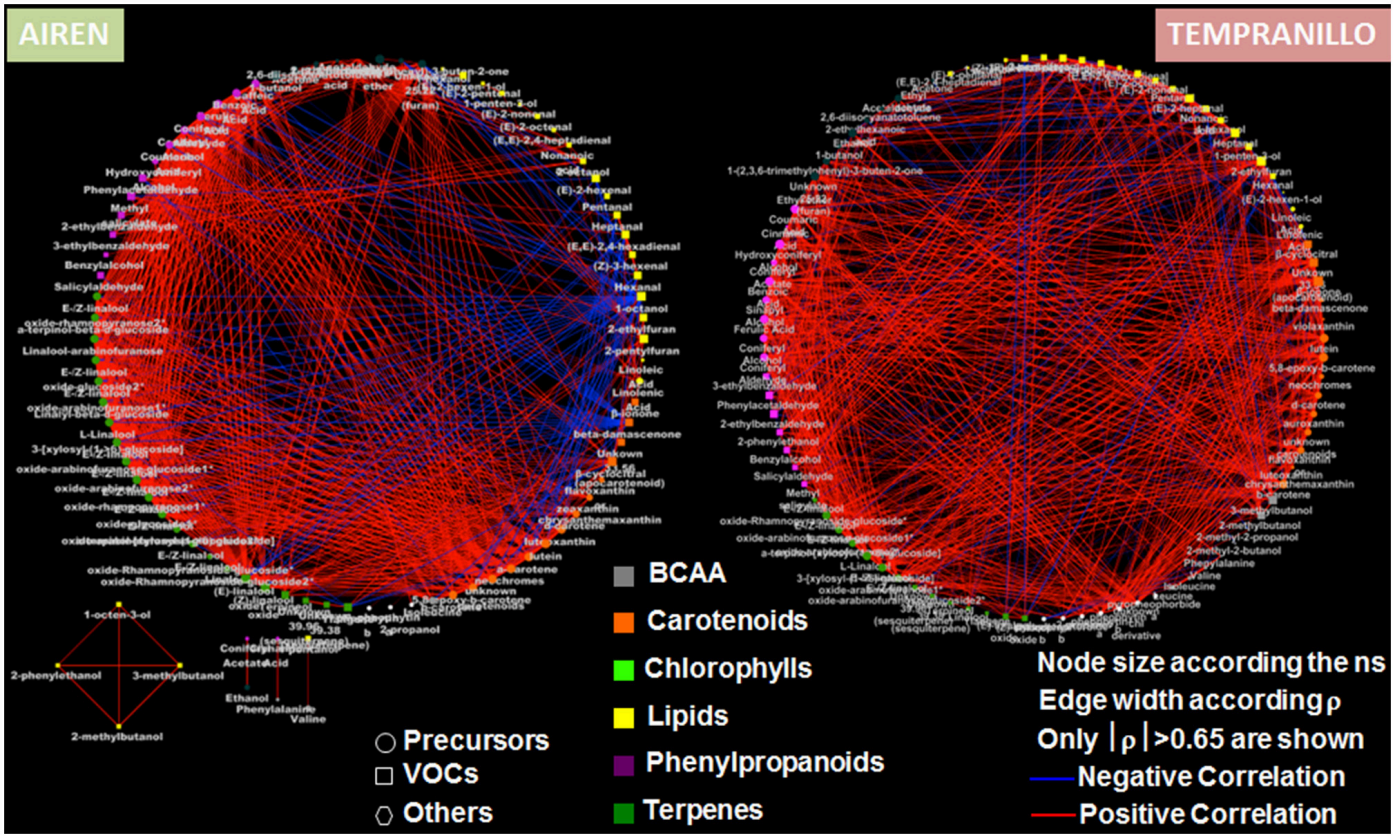
**Correlation networks of precursor and volatile metabolites in ripening berries of Airen and Tempranillo grape varieties**. Networks are visualized as circles with nodes of different sizes according the node strength (ns). Lines joining the nodes represent postive (red) and negative (blue) correlations, of width proportional to each corresponding |ρ|.

Furthermore, we used correlative analyses to build two correlation networks, a different approach to investigate relationships among the different metabolic pathways, as well as within the same metabolism (Figure 3). In agreement with what was previously observed, the two varieties mainly differentiate at the lipid level, yielding a negative correlation region in the Airén berries. Additionally, we observed the presence of areas of high positive correlations (e.g., number of nodes belonging to the same metabolic pathways harvesting a large number of correlations >|0.65|) like the terpene and carotenoid pathways (for Airén) and the phenylpropanoid metabolism (both varieties, but with a greater number of correlations in Airén compared to Tempranillo). The distinct pattern of the lipid precursors and volatiles with respect to the other metabolic classes and between the two varieties prove that the volatile evolution in the Tempranillo berries occurs through a concerted process in which metabolites from the different pathways move together (general presence of positive correlations). On the contrary, Airén berries display a “metabolite imbalance” between the primary (lipids, negatively correlated toward all the other pathways) and the secondary (phenylpropanoids, carotenoids, terpenes, which are positively correlated) metabolism. Finally, the evaluation of the “node strength” (ns) (Diretto et al., 2010) which is the average of all the |ρ| of a node, demonstrates that, with the exception of the primaries as well as some other random metabolite, in general all the compounds under investigation take part with the same “weight” in the metabolic shift arising during berry ripening.

To gain insights into the biological roles played by *V. vinifera* GH Family 1, LOX, HPL, OMT, ADH, and CCD, a set of primers have been designed based on the full length sequences obtained at the Genoscope database with the exception of ADH and LOX, where only 3 sequences per gene were selected (Supplementary Table 1) for OMT. Using these primers, qRT-PCR analyses were carried out to determine the expression pattern in both Tempranillo and Airén cultivars, using tissues from the 10 stages.

**Figure 4:**
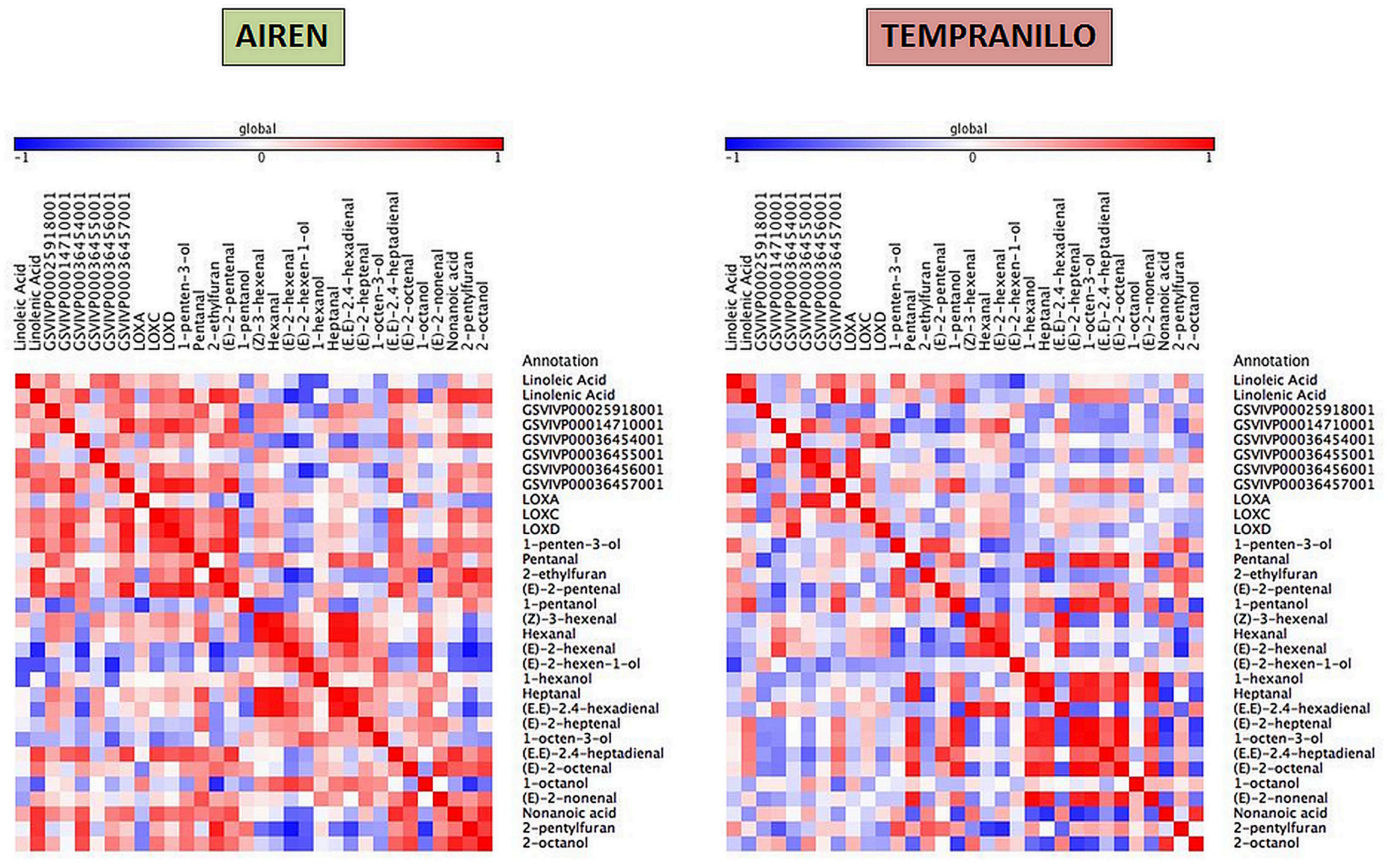
**Heatmap plots for gene-metabolite relationships in lipid biosynthesis**. Legend on the right indicates the corresponding names of genes and metabolites. Red and blue shaded boxes indicate, respectively, different extents of positive and negative correlations; white boxes indicate no correlation.

**Figure 5:**
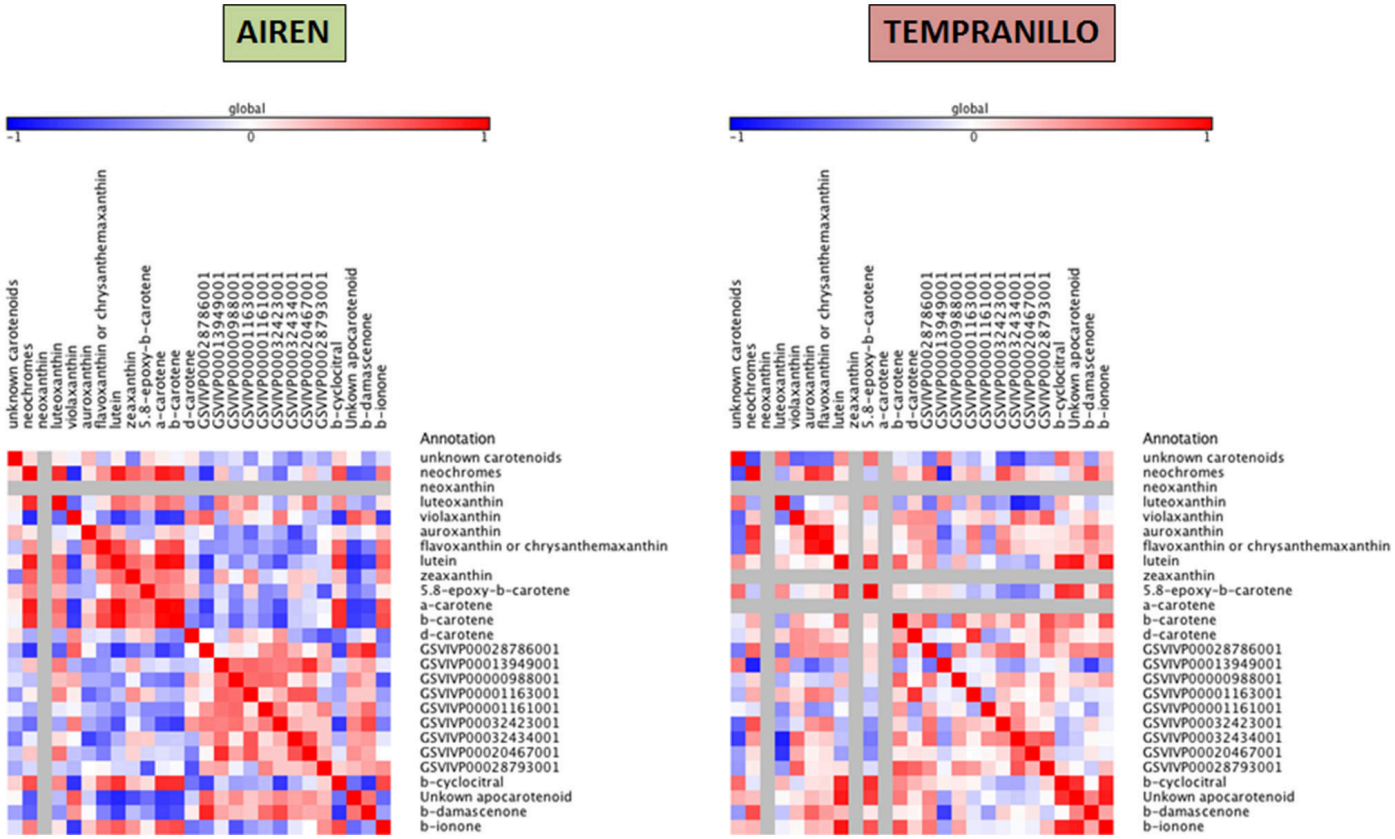
**Heatmap plots for gene-metabolite relationships in carotenoid biosynthesis**. Legend on the right indicates the corresponding names of genes and metabolites. Red and blue shaded boxes indicate, respectively, different extents of positive and negative correlations; white boxes indicate no correlation.

**Figure 6:**
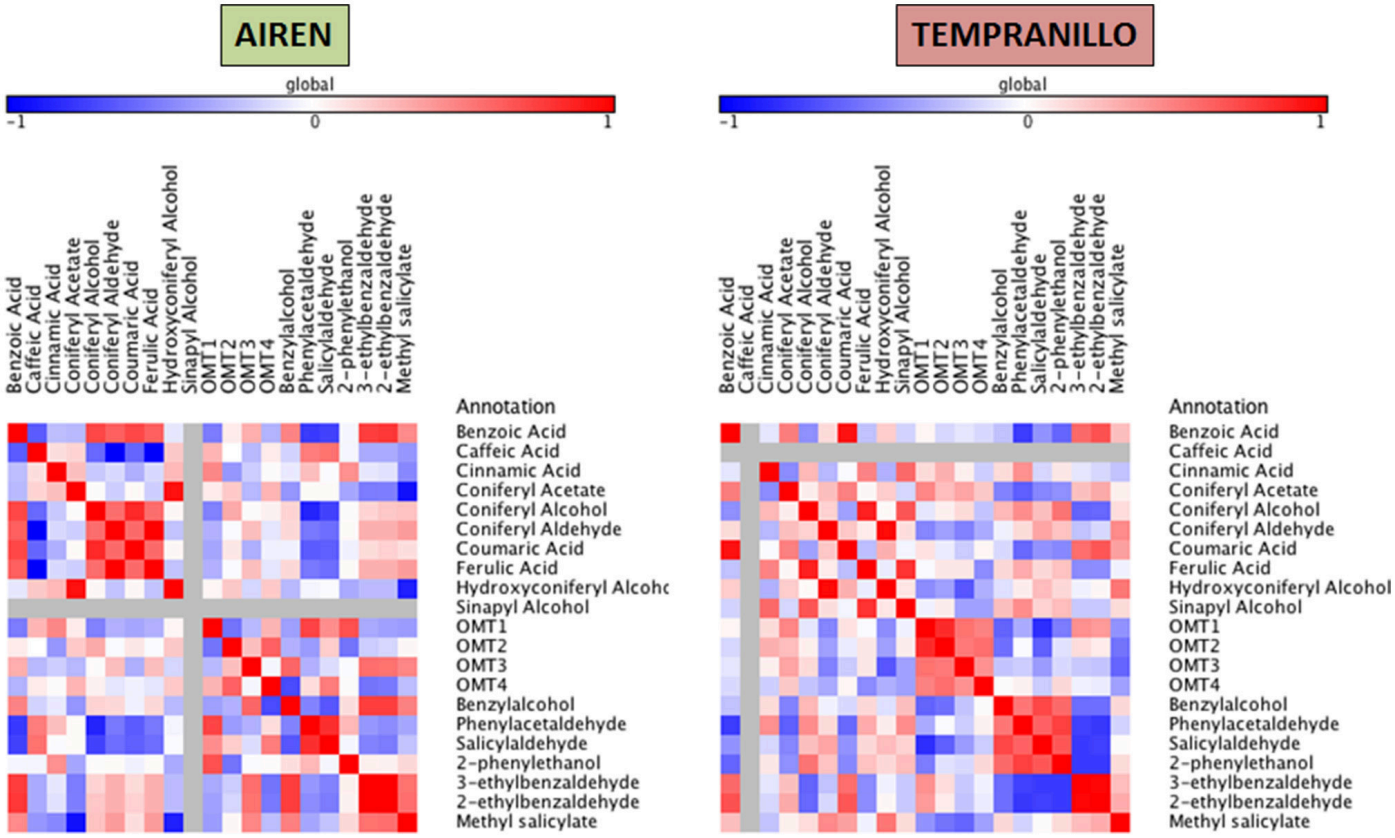
**Heatmap plots for genes-metabolites relationships in phenylpropanoid biosynthesis**. Legend on the right indicates the corresponding names of genes and metabolites. Shades of red and blue colored boxes indicates, respectively, different extents of positive and negative correlations; white boxes refer indicate no correlation.

Taking together the expression analyses and the metabolite compound levels throughout the different stages, for each variety we have built three heat maps referring to lipid, carotenoid and phenylpropanoid metabolism (Figures 4–6, respectively). To achieve this, we used Pearson correlation analysis, a best-fit approach that creates a mathematical simulation of expression values using the available experimental data. Significant correlation does not necessarily mean that there is a cause-effect relationship between genes and metabolites; although it allowed us to suggest possible candidates for a gene function, and also to discard genes as unrelated to metabolites.

For each pathway, elements are placed according to the following order: non-volatile precursors, genes (LOX, HPL, CCD, and OMT) and volatile compounds. Using this approach, it is possible to identify metabolites and transcripts whose levels show a concordant or opposite evolution, resulting in, respectively, positive and negative correlations. Since GH 1 and ADH proteins have low substrate affinities, we have built a correlation network for assessing their putative activities (Supplementray Figure 3).

Overall correlation values between volatile, non-volatile levels and gene expression were higher in the Airén variety than in Tempranillo (Supplementary Tables 3–5).

Regarding the lipid metabolism as shown in Figure 4, linolenic acid showed a higher significant positive relationship with some volatiles than linoleic acid as (E,E)-2,4-heptadienal (0.75), 1-penten-3-ol (0.80), 2-ethylfuran (0.84), (E)-2-pentenal (0.75), nonanoic acid (0.81), 2-pentylfuran (0.80), and 2-octanol (0.75) while both fatty acids correlated negatively with (E)-2-hexen-1-ol (−0.68). GSVIVP00014710001 (HPL2) and GSVIVP00036457001 (HPL6) correlated positively between each other (0.90) and with LOXD (0.85, 0.91), respectively; LOXA correlated positively with (Z)-3-hexenal (0.70) and negatively with 2-pentylfuran (−0.70), whereas LOXC and LOXD were strongly correlated to each other (0.87). LOXC correlated strongly with GSVIVP00014710001 (HPL2) (0.69), GSVIVP00036456001 (HPL5) (0.71) and GSVIVP00036457001 (HPL6) (0.81).

In Tempranillo, more correlations among the volatile compounds were found than with the precursors or HPL and LOX genes, for example (E,E)-2,4-hexadienal and (Z)-3-hexenal have a strong correlation (0.98) with each other. Only linoleic acid correlated positively with GSVIVP00036457001 (HPL6) (0.70). A low relationship was obtained between (E)-2-hexenal and GSVIVP00014710001 (HPL2) (0.67) and between (E)-2-pentenal and LOXA. Transcript levels of LOXD and HPL6 correlate negatively with linolenic acid (-0.74) and positively with linoleic acid (0.76), respectively.

Four LOX genes VvLOXA, VvLOXO, VvLOXC, and VvLOXD from the white grape cultivar Sauvignon Blanc have been isolated and characterized (Podolyan et al., 2010). The recombinant LOXA-TP and LOXO-TP proteins have been expressed and both enzymes were able to convert LnA into 13(S)-hydroperoxyoctadecatrienoic acid and LA into 13(S)- hydroperoxyoctadecadienoic acid. During berry development for three seasons, transcripts from VvLOXA exhibited an initial decrease in early stages of development, followed by an increase in expression around veraison. LOX enzymes generate some derivatives that can be catalyzed by hydroperoxide lyase to produce aldehydes. Our data suggested that linoleic and linolenic are catalyzed by LOXA and HPL2, producing (E)-2-hexenal and (E)-2-pentenal, whhile it is possible that HPL6 could also be involved in this pathway.

Correlations from carotenoid metabolism exhibited more relationships among the precursors and volatile compounds than CCD expression genes. Different patterns were found in Airén and Tempranillo. In the white grape cultivar, β-cyclocitral positively correlated with neochromes (0.70), lutein (0.81), β-carotene (0.82), and α-carotene (0.83). β-damascenone correlated with GSVIVP00028786001 (CCD1) (0.86) and also, to a lesser extent with GSVIVP00032423001 (CCD1-1) (0.73).

In the red grape cultivar, looking at the relationships between precursors and VOCs, positive correlations were found among the unknown 33.56 apocarotenoid (0.91, 0.77), β-cyclocitral (0.87, 0.82), and β-ionone (0.92, 0.68) with lutein and 5,8-epoxy-β-carotene respectively. β-damascenone showed a low correlation with neochromes (0.71). On the other hand, positive correlations between gene expressions and precursors were found only between δ-carotene and GSVIVP00001163001 (CCD4a) (0.81) and between neochromes.

Our data showed a relationship among CCD1, lutein, β-ionone, β-cyclocitral, β-damascenone, and the unkown 33.56 apocarotenoid. It is known that CCD1 enzymes are involved in the cleavage of the 5,6 (5′,6′) (Vogel et al., 2008); 7,8 (7′,8′) (Ilg et al., 2009) and 9,10 (9′,10′) (Schwartz et al., 2001) double bonds to produce a variety of volatiles. In grape, VvCCD1 is able to produce 3-hydroxy-β-ionone from zeaxanthin (Mathieu et al., 2005), pseudoionone from lycopene, β-ionone from β-carotene and 6-methyl-5-heptene-2-one (6MHO) from lutein (Lashbrooke et al., 2013). β-damascenone is generated from multiple grape glycoconjugated precursors as lutein (Pineau et al., 2007). *In vitro* enzyme assay was carried out by (Mathieu et al., 2005) using VvCCD1 from *V. vinifera* L. cv Shiraz catalyzed only the cleavage of zeaxanthin and lutein to produce 3-hydroxy-β-ionone but not β-carotene as a substrate. However VvCCD1 isolated from *V. vinifera* L. cv Pinotage by (Lashbrooke et al., 2013), was capable of catalyzing the cleavage of lycopene, β-carotene and ε-carotene, but not neurosporene and ζ-carotene. Recent studies suggest that apocarotenoids instead of carotenoids act as the major substrates of CCD1 in plant (Floss et al., 2008; Ilg et al., 2010; Rubio-Moraga et al., 2014).

No clear associations were found among the precursors and the CCD4 genes in either the white or in the red cultivars. The CCD4 family contains at least two forms of genes with different structure and genome position (Ahrazem et al., 2010). The main group contains enzymes with a 9,10 (9′,10′) double bond cleavage activity (Rubio et al., 2008; Huang et al., 2009) and a second clade with 5,6 (5′,6′) activity as CCD4a and b enzymes from *V. vinifera* (Lashbrooke et al., 2013). A new CCD4 from citrus was recently isolated and has the ability to cleave asymmetrically at the 7′,8′ double bond in zeaxanthin and β-cryptoxanthin (Rodrigo et al., 2013). In grape, VvCCD4a and VvCCD4b were able to produce α-ionone from ε-carotene and geranylacetone from neurosporene, also VvCCD4a and VvCCD4b were capable of releasing 6-MHO from lycopene and geranylacetone from ζ-carotene (Lashbrooke et al., 2013). Despite the low correlation among lutein and the CCD4 a and b, the plastidial location of these CCDs and the characterization of an orthologue from saffron CsCCD4c, with a restricted expression in stigmas, having activity 9,10 (9′,10′) over lutein (Rubio-Moraga et al., 2014) suggest that these enzymes might use lutein as a substrate.

The fact that β-cyclocitral was detected in both cultivars indicate the presence of a CCD4 which cleaves at the 7′,8′ double bond in zeaxanthin or lutein. Two more VvCCD4c and d were described in *Vitis* showing a 97% of identity in nucleotides between each other and were related to CcCCD4b from citrus and PtCCD4c and d from *Populous truncata* (Ahrazem et al., 2010). Even though the expression of the VvCCD4c could not be detected in any of the tissues analyzed by (Lashbrooke et al., 2013), these enzymes seem to be candidates to release β-cyclocitral from zeaxanthin or lutein by an asymmetric cleavage.

Concerning the phenylpropanoid pathway, different patterns were obtained in both cultivars. In Airén, a cluster formed by benzoic acid, coniferyl alcohol (0.73) and coumaric acid (0.72) correlated positively among each other and also with the volatiles 3- and 2-benzaldehyde (0.76 and 0.79). Coniferyl acetate showed strong positive and negative correlations with hydroxyconiferyl alcohol (0.95) and methyl salicylate (-0.92) respectively. OMT1 has a relatively high correlation with phenylacetaldehyde (0.74) and in a lesser extent with 2-phenylethanol (0.69), OMT4 has a negative association with benzylalcohol (−0.70). Some volatiles showed relationships among each other as benzyalcohol and 3- and 2-ethylbenzaldehyde (0.76 and 0.76).

A strong positive relation was found between benzoic acid and coumaric acid (0.93) with 2-ethylbenzaldehyde (0.68). Another cluster is formed by coniferyl alcohol, ferulic acid and sinapyl alcohol, which are all strongly correlated among themselves (up to 0.88). High positive correlation was also found between coniferyl aldehyde and hydroxyconiferyl alcohol (0.97). OMT1 and OMT2 were related to each other and were also negatively associated with salicylaldehyde (0.81). We were not able to establish any relationship among the OMTs studied and the VOC compounds.

Regarding branched-chain amino acids and the volatile compounds related to them, no significant correlation appeared either in Airén or in Tempranillo.

In relation to GH 1 (Supplementary Table 5 and Supplementary Figure 3), a cluster formed by GS6, GS21, and GS25 showed a positive correlation among each other and showed the same pattern against the metabolites involved in the amino acid metabolism (precursors: Isoleucine, leucine, and valine; volatiles: 2-methyl-2-propanol, 3-methylbutanol and 2-methylbutanol) and against two lipids [linolenic acid and (Z)-3-hexenal) and a terpene (Unknown 39.38 (sesquiterpene)], suggesting a putative role in the generation of these volatiles. Similarly, GS9 also exhibited a broad set of significant correlations toward metabolites of the lipid pathway, positive with linoleic acid (0.66), 1-penten-3-ol (0.77), and 2-ethylfuran (0.74), and negative with Hexanal and (E)-2-hexenal (−0.68 each), which could, thus, let hypothesize a function in the evolution of the lipid-derived volatiles.

GS10, GS15, GS24, and GS28 placed, together with the already mentioned GS9, in the most crowded region of the network, and showed significant positive correlations with almost all the phenylpropanoid precursors detected with score values ranging from 0.91 (Coniferyl Aldehyde) to 0.67 (Coniferyl Alcohol) and also with some terpene glucosides (α-terpinol-[xylosyl-(1 → 6)-glucoside] (0.82); L-Linalool 3-[xylosyl-(1->6)-glucoside] (0.78); E-/Z-linalool oxide-arabinofuranose1^*^ (0.82); E-/Z-linalool oxide-arabinofuranose-glucoside1^*^ (0.84); E-/Z-linalool oxide-arabinofuranose-glucoside2^*^ (0.88)). Surprisingly, these transcripts also displayed significant positive correlations with carotenoid precursors and apocarotenoid compounds [for instance, auroxanthin (0.92) and flavoxanthin (0.84) in the former, and b-ionone and the Unknown 33.56 (apocarotenoid) (0.82 each)].

Transcript levels of glycosidases GS1, GS5, GS12, GS16, GS20, GS22, and GS26 showed no significant correlation with any volatile compound. Therefore, the proteins encoded by these genes would not be expected to be involved in the production of any of the volatile compounds identified, at least in the wide range of developmental stages studied, or unless their activities would be mostly regulated at translational or post-translational level. The rest of glycosidases not mentioned above have few correlations with the metabolites detected (for instance, GS8 with limonene or GS23 with (E)-2-pentenal), indicating either a very specialized activity, or that these glycosidases should produce a minor effect in the generation of the final bouquet.

In the classic model of volatile emission, the occurrence of significant negative correlations between the precursor metabolite and the genes coding for volatile-producing enzymes would be expected, along with a positive correlation between the latter and the volatiles. However, this simplified model does not take into consideration regulation phenomena at protein/enzymatic activity level (as mentioned above). Additionally, it must be remarked that some precursors (especially the ones involved in primary metabolism) are needed at high constitutive levels for a series of additional functions in cell metabolism, or are accumulated as sink (as in the case of the terpene glucosides). In this complex framework, we believe that the presence of significant correlations, although with an opposite sign with respect to the expected, could be biologically relevant, and could reflect the presence of specific metabolic tunings.

ADH1 exhibited only a few correlations with (E)-2-hexen-1-ol (0.67) and, contrary to the expectation, with the pyropheophorbide a (0.71), while ADH2 and ADH3 displayed almost the same pattern against the metabolites and showed high correlative power toward lipid derivatives (Supplementary Table 5). However, ADH3 showed higher scores than ADH2 and more relationships with other metabolites such as (E)-2-hexenal (0.67), 3-ethylbenzaldehyde (0.84), 2-ethylbenzaldehyde (0.74), terpeniol (0.73) and 2,6-diisocyanatotoluene (0.82). It has been shown that grape ADH1 gene expression was detected in the first phase of fruit development, while ADH2 has been described as a berry ripening-related isogene, with data suggesting that transcriptional regulation of these genes and ADH enzyme activity could partially be related to the ethylene signaling pathway (Cirilli et al., 2012).

Our data allow a general and extensive view of the evolution of volatile and non-volatile compounds from the early formation of the berry to the post ripening stages, along with their relationships with some transcripts involved in their biosynthesis (Figure 3). Differences between the two varieties regarding VOCs were found, even though these variations were mostly attributed to differences in the levels of the substances that constitute grape aroma rather than to qualitative differences in the volatile compounds produced. The results obtained provide potential glycosidase candidates that could participate in the final aroma of Airén and Tempranillo, such as GS9, GS10, GS15, GS16, GS21, GS24, and GS25. In relation to lipid metabolism, data showed the possible involvement of LOXA and HPL2 to generate (E)-2-hexenal and (E)-2-pentenal. At the carotenoid metabolism level, volatiles exhibited a higher extent of correlations toward their precursors compared to the biosynthetic genes; although a notable exception was represented by CCD1, which was related mainly with the production of β-ionone and CCD4c, and seems to be the candidate for the release of β-cyclocitral from zeaxanthin or lutein by an asymmetric cleavage. Concerning phenylpropanoid and branched-chain amino acid pathways, no clear relationships were found among the metabolites and gene expression. Interestingly, the white variety showed a higher “metabolite imbalance” between primary (lipids, negatively correlated toward all the other pathways) and secondary (phenylpropanoids, carotenoids, terpenes, which are positively correlated among each other) metabolism than the red variety. Furthermore, correlation analysis also showed a higher degree of overall correlation in precursor/volatile metabolite-metabolite levels in Airén, which confirms a distinct mechanism of the white varieties for producing an enriched aroma bouquet compared to the red ones.

## Author contributions

OA, LG conceived and designed the experiments with the help of AG, GD. JR, AT performed the volatile detection and quantification experiments. AT, AR, and LG contributed to the preparation of the RNA samples and performed the qRT-PCR experiments. GD, AG performed the precursors detection and quantification analyses. OA, JR, and GD achieved the *in silico*, statistical, and bioinformatics analyses. OA, GD, JR, and LG wrote the manuscript and all authors contributed to the discussion and approved the final manuscript.

### Conflict of interest statement

The authors declare that the research was conducted in the absence of any commercial or financial relationships that could be construed as a potential conflict of interest.
