# Supplementary material for: Gene-Metabolite Networks of Volatile Metabolism in Airen and Tempranillo Grape Cultivars Revealed a Distinct Mechanism of Aroma Bouquet Production
Source: Front Plant Sci. 2016 Oct 27;7:1619. doi: 10.3389/fpls.2016.01619 (PMC5082229; doi:10.3389/fpls.2016.01619)
Supplement: Supplementary file 1 [file Presentation1.PDF]

Supplementary Material

Gene-metabolite networks of volatile metabolism in Airen and Tempranillo grape cultivars revealed a distinct mechanism of aroma bouquet production

José L. Rambla, Almudena Trapero-Mozos, Gianfranco Diretto, Angela Rubio-Moraga, Antonio Granell, Lourdes Gómez-Gómez, Oussama Ahrazem

*** Correspondence:** Oussama Ahrazem, oussama.ahrazem@uclm.es

Supplementary Figures and Tables

1.1 Supplementary Figures

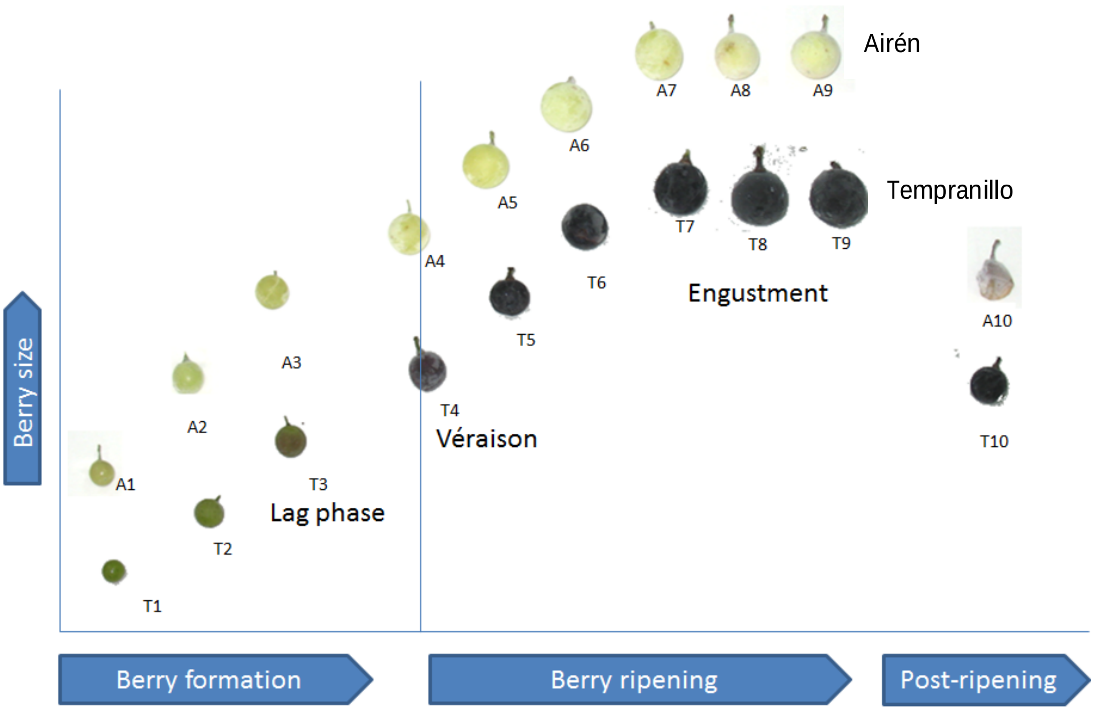

Supplementary Figure 1. The major phases (lag phase, véraison and engustment) of berry development and ripening of Airén and Tempranillo grape berries. A1 to A10 and T1 to T10 correspond to the different stages analysed during Airén and Tempranillo development, respectively.

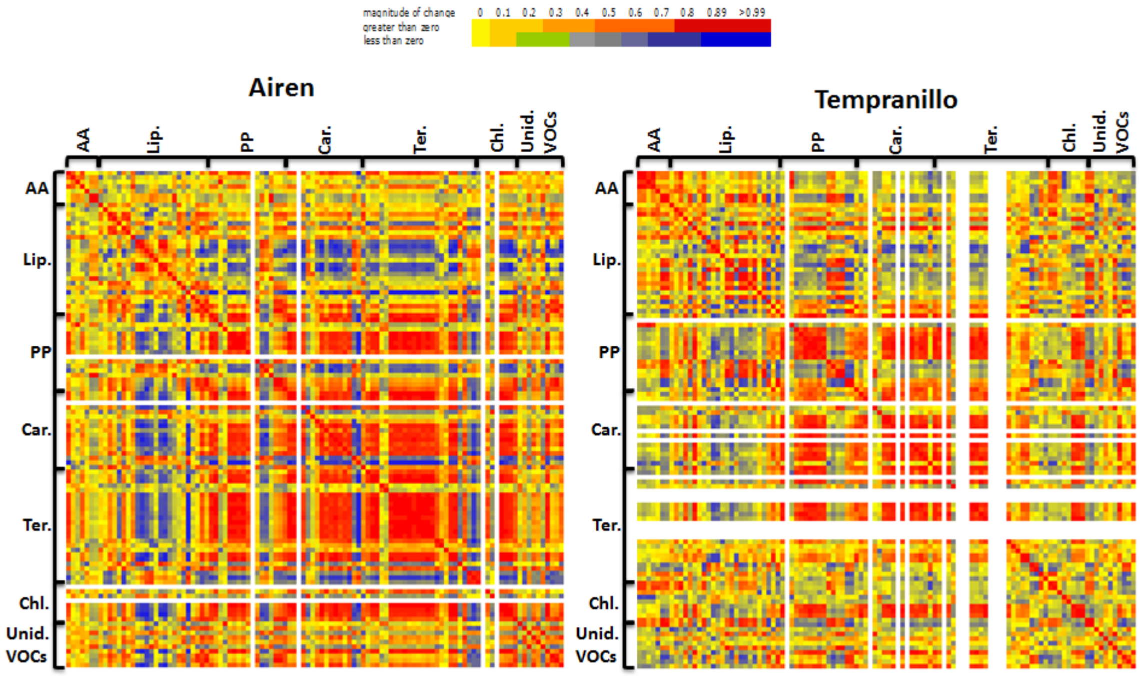

Supplementary Figure 2. Metabolite-metabolite correlation matrix in ripening berries of Airen and Tempranillo grape varieties. Each square represents the Pearson correlation coefficient between the metabolite heading the column with the metabolite heading the row. Hues of red and blue define, respectively, the strength of the positive and negative correlation. For details, see materials and methods.

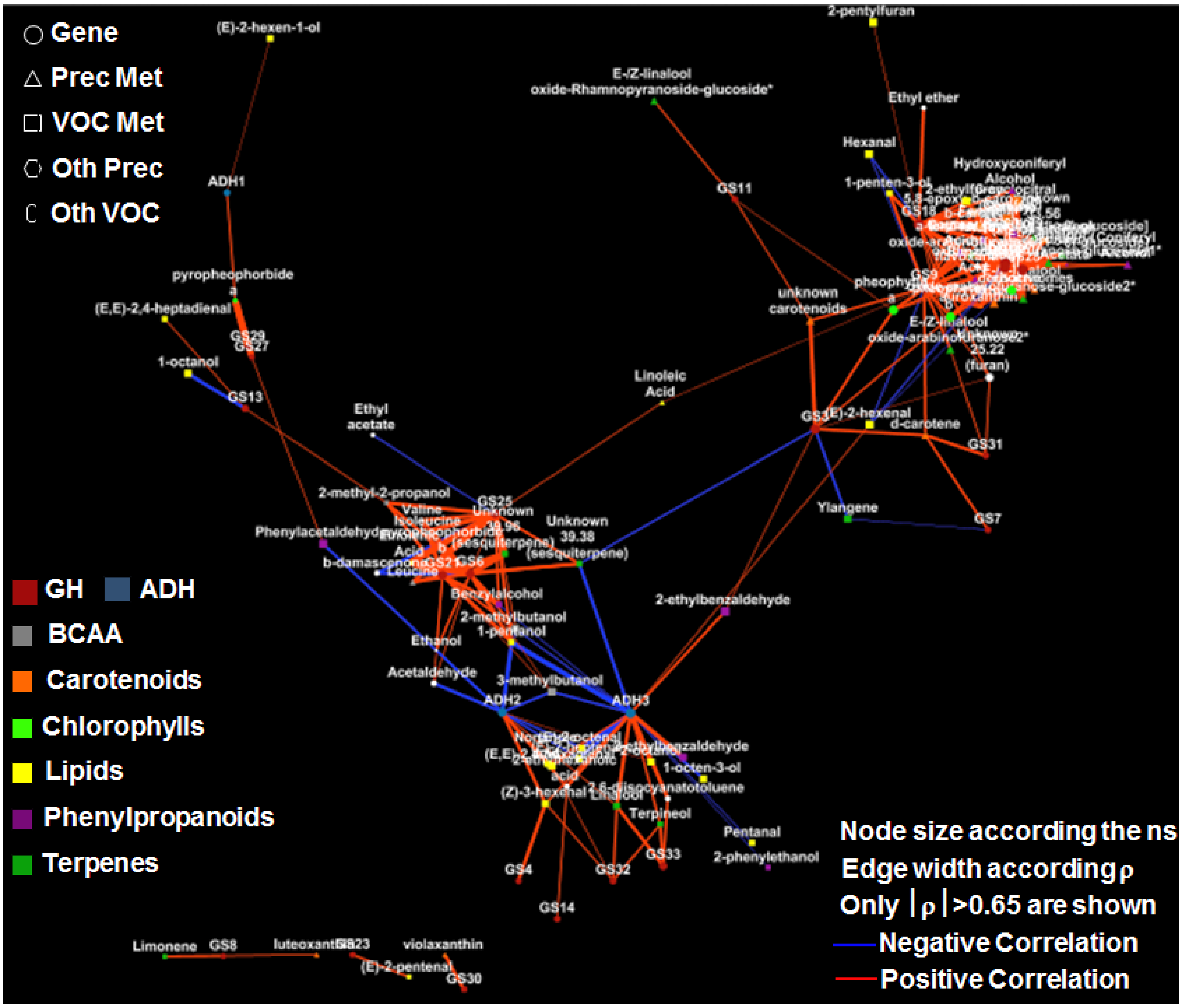

Supplementary Figure 3. Correlation networks for assessing GH and ADH putative activities in Airén and Tempranillo. Only correlations >|0.65| are shown. Red and blue edges indicate, respectively, positive and negative correlations, and edge width is proportional to |ρ|. Detailed correlation coefficients used to generate the networks are available in Supplementary Table 5.

1.2 Supplementary Tables

Supplementary Table 1. Primer sequences used for quantitative real-time PCR.

Supplementary Table 2. Volatiles compounds detected in Airén and Tempranillo using Headspace solid phase microextraction (HS-SPME) technique during 2010 and 2011.

Supplementary Table 3. Pearson correlation coefficients of precursor-, volatile-metabolites and genes under investigation, classified according the different metabolism (lipids, carotenoids, phenylpropanoids) in ripening berries of Airen grape variety. Red and Green mark, respectively, positive and negative correlations (|r|>0.65).

Supplementary Table 4. Pearson correlation coefficients of precursor-, volatile-metabolites and genes under investigation, classified according the different metabolism.

Supplementary Table 5. Pearson correlation coefficients calculated, for each GS and ADH gene, against all the precursor and volatile metabolites. Red and Green mark.
